# Bidirectional Mendelian randomization and mediation analysis of million-scale data reveal causal relationships between thyroid-related phenotypes, smoking, and lung cancer

**DOI:** 10.7555/JBR.38.20240421

**Published:** 2025-03-10

**Authors:** Xiang Wang, Xuan Wang, Mengsheng Zhao, Lijuan Lin, Yi Li, Ning Xie, Yanru Wang, Aoxuan Wang, Xiaowen Xu, Can Ju, Qiuyuan Chen, Jiajin Chen, Ruili Hou, Zhongwen Zhang, David C. Christiani, Feng Chen, Yongyue Wei, Ruyang Zhang

**Affiliations:** 1 Department of Biostatistics, Center for Global Health, School of Public Health, Nanjing Medical University, Nanjing, Jiangsu 211166, China; 2 China International Cooperation Center for Environment and Human Health, Nanjing Medical University, Nanjing, Jiangsu 211166, China; 3 Jiangsu Key Lab of Cancer Biomarkers, Prevention and Treatment, Cancer Center, Collaborative Innovation Center for Cancer Personalized Medicine, Nanjing Medical University, Nanjing, Jiangsu 211166, China; 4 Department of Biostatistics, University of Michigan, Ann Arbor, MI 48109, USA; 5 Pulmonary and Critical Care Division, Department of Medicine, Massachusetts General Hospital and Harvard Medical School, Boston, MA 02114, USA; 6 Department of Environmental Health, Harvard T.H. Chan School of Public Health, Boston, MA 02115, USA; 7 Center for Public Health and Epidemic Preparedness & Response, Peking University, Beijing 100191, China; 8 Changzhou Medical Center, Nanjing Medical University, Changzhou, Jiangsu 213164, China

**Keywords:** hypothyroidism, hyperthyroidism, lung neoplasms, smoking, causality, Mendelian randomization analysis

## Abstract

Emerging evidence highlights the role of thyroid hormones in cancer, although findings are controversial. Research on thyroid-related traits in lung carcinogenesis is limited. Using UK Biobank data, we performed bidirectional Mendelian randomization (MR) to assess causal associations between lung cancer risk and thyroid dysfunction (hypothyroidism and hyperthyroidism) or functional traits (free thyroxine [FT4] and normal-range thyroid-stimulating hormone [TSH]). Furthermore, in the smoking-behavior-stratified MR analysis, we evaluated the mediating effect of thyroid-related phenotypes on the association between smoking behaviors and lung cancer. We demonstrated significant associations between lung cancer risk and hypothyroidism (hazard ratio [HR] = 1.14, 95% confidence interval [CI] = 1.03–1.26, *P* = 0.009) and hyperthyroidism (HR = 1.55, 95% CI = 1.29–1.87, *P* = 1.90 × 10^−6^) in the UKB. Moreover, the MR analysis indicated a causal effect of thyroid dysfunction on lung cancer risk (OR_inverse variance weighted [IVW]_ = 1.09, 95% CI = 1.05–1.13, *P* = 3.12 × 10^−6^ for hypothyroidism; OR_IVW_ = 1.08, 95% CI = 1.04–1.12, *P* = 8.14 × 10^−5^ for hyperthyroidism). We found that FT4 levels were protective against lung cancer risk (OR_IVW_ = 0.93, 95% CI = 0.87–0.99, *P* = 0.030). Additionally, the stratified MR analysis demonstrated distinct causal effects of thyroid dysfunction on lung cancer risk among smokers. Hyperthyroidism mediated the effect of smoking behaviors, especially the age of smoking initiation (17.66% mediated), on lung cancer risk. Thus, thyroid dysfunction phenotypes play causal roles in lung cancer development exclusively among smokers and act as mediators in the causal pathway from smoking to lung cancer.

## Introduction

Lung cancer is the leading cause of cancer-related mortality and the second most common cancer in the United States, with approximately 238000 new cases and 127000 deaths in 2023^[1]^. The obvious clinical symptoms of lung cancer often appear in the late stages of the disease, so most patients are not diagnosed until the disease has advanced^[2–3]^, which is accompanied by a poor prognosis and a low five-year survival rate^[4]^. As the burden of lung cancer increases, it is critically important to understand the underlying pathogenesis, clarify the associated etiology, and identify potentially modifiable risk factors to improve disease prevention.

Growing evidence has established the crucial role of thyroid hormones in regulating physiological processes of tumor cell proliferation, differentiation, and metabolism^[5–7]^. Therefore, thyroid dysfunction, characterized by decreased (hypothyroidism) or increased (hyperthyroidism) secretion of thyroid hormones, may be involved in carcinogenesis^[8]^ and is considered a potential and preventable cancer risk factor^[9–10]^. Previous studies have investigated the association between thyroid dysfunction and cancer risk, but the findings are conflicting^[11]^. For example, a prospective cohort study in Western Australia found no association between lung cancer risk and thyroid function phenotypes, including thyroid-stimulating hormone (TSH) and free thyroxine (FT4)^[12]^, which contradicts positive results from a Rotterdam study^[10]^. Additionally, tobacco smoking is the most common established cause of lung cancer. Population-based studies have suggested that tobacco smoking is also associated with thyroid function phenotypes^[13–14]^, with smokers exhibiting lower levels of TSH and higher levels of thyroid hormone^[15–16]^. Thus, it is necessary to determine whether there is a causal association between thyroid-related phenotypes (thyroid dysfunction, including hypothyroidism and hyperthyroidism, as well as thyroid function phenotypes, such as FT4 and TSH) and lung cancer risk, and to determine how thyroid-related phenotypes mediate the effect of smoking on lung cancer risk.

Despite the many advantages of observational studies, their limitations are well recognized^[17]^, because they are vulnerable to potential confounding factors, measurement errors, and reverse causality. These issues hinder the ability to draw causal inferences about the association between thyroid-related phenotypes and cancer risk. Mendelian randomization (MR) studies, which use genetic variants as instrumental variables (IVs) to establish causal relationships between exposures (thyroid-related phenotypes) and outcomes (lung cancer risk), are known to be less vulnerable to bias than traditional observational studies^[18]^. Because genetic alleles are randomly assigned at conception, the MR analyses are not influenced by reverse causation^[19]^.

In the current study, we performed an observational analysis to characterize the association between thyroid dysfunction (hypothyroidism and hyperthyroidism) and lung cancer risk using data from the UK Biobank (UKB). Furthermore, we determined the causal associations between thyroid-related phenotypes and lung cancer risk, as well as the interaction between thyroid-related phenotypes and smoking in association with lung cancer risk using a two-sample MR analysis. Additionally, we assessed the mediating role of thyroid-related indices in the association between smoking behavior and lung cancer risk.

## Materials and methods

### Data resources and study populations

#### Individual-level data of thyroid phenotypes, smoking phenotypes, and lung cancer from the UKB

UKB is a large prospective study that assessed over 500000 participants aged from 40 to 70 years in 22 centers in the UK between 2006 and 2010 at baseline. Details of the study protocol have been previously described^[20]^. The UKB data are available through an application at https://www.ukbiobank.ac.uk/. Genetic data are available for 487409 participants. Lung cancer cases were collected based on the ICD-10 and ICD-9 diagnostic codes (C33 and 162.X, respectively) or self-reported lung cancer histology (Field ID: 20001). Hypothyroidism and hyperthyroidism cases were collected based on the ICD-10 and ICD-9 diagnostic codes (Hypothyroidism: E03.9 and 244.X; Hyperthyroidism: E05.9 and 242.X) or self-reported non-cancer illness (Field ID: 20002).

We enrolled 500714 participants in the final analysis, including 5119 participants (1.02%) diagnosed with lung cancer (age, 61.58 [± 8.10] years; body mass index [BMI], 27.41 [± 4.78] kg/m^2^; 2615 [51.08%] male) and 495595 participants (98.98%) without lung cancer (age, 56.46 [± 5.95] years; BMI, 27.43 [± 4.80] kg/m^2^; 225424 [45.49%] male). Additionally, participants diagnosed with lung cancer had a higher incidence of hypothyroidism (479 [9.36%]) and hyperthyroidism (131 [2.56%]), compared with those in the control group (***Supplementary Table 1*** [available online]), while the data on FT4 and TSH were not available in UKB.

#### Summary-level data from GWAS of thyroid-related phenotypes from meta-analysis

We obtained the summary-level data from GWAS of thyroid-related phenotypes, including thyroid dysfunction for hyperthyroidism and hypothyroidism, as well as thyroid-related phenotypes for FT4 and TSH. Specific protocols for the studies contributing to the meta-analysis were described previously^[21–22]^. Briefly, significant SNPs associated with hypothyroidism (38624 cases/76464 controls) and hyperthyroidism (6160 cases/301639 controls) were extracted from the FinnGen project (Release 7). The SNPs associated with the circulating FT4 levels were determined from a meta-analysis of GWAS results, including 19 cohorts with 49269 subjects of European ancestry in the ThyroidOmics Consortium^[23]^. The TSH-related SNPs were determined from the largest meta-analysis of GWAS conducted to date by Zhou *et al*^[24]^, which included the HUNT study (*n* = 55342), the MGI biobank (*n* = 10085), and the ThyroidOmics Consortium (*n* = 54288).

#### Summary-level data from GWAS of smoking phenotypes from meta-analysis

Summary-level data from GWAS of four smoking phenotypes, including smoking initiation, smoking cessation, cigarettes per day, and the initiation age of regular smoking, were obtained from a meta-analysis of 60 cohorts with up to 2.6 million individuals of European ancestry^[25]^. Briefly, the meta-analysis was performed centrally using rareGWAMA, and sample sizes were 2669029 for smoking initiation, 1147272 for smoking cessation, 618489 for cigarettes per day, and 618541 for the initiation age of regular smoking (***Supplementary Table 2***, available online). To capture smoking heaviness, duration, and cessation, a lifetime smoking index was constructed using 462690 individuals from the UKB^[26]^. In brief, we combined the smoking measures into a lifetime smoking index along with a simulated half-life (*τ*) constant.

#### Summary-level data from GWAS of lung cancer from meta-analysis

Summary-level data from GWAS of lung cancer were obtained from a meta-analysis of populations in the UKB, Transdisciplinary Research Into Cancer of the Lung (TRICL), and International Lung Cancer Consortium (ILCCO), including 34056 lung cancer cases and 470856 controls^[27]^. Subgroup analyses, as well as smoking status and lung cancer histology, are shown in ***Supplementary Table 3*** (available online).

### Statistical analysis

**Figure 1:**
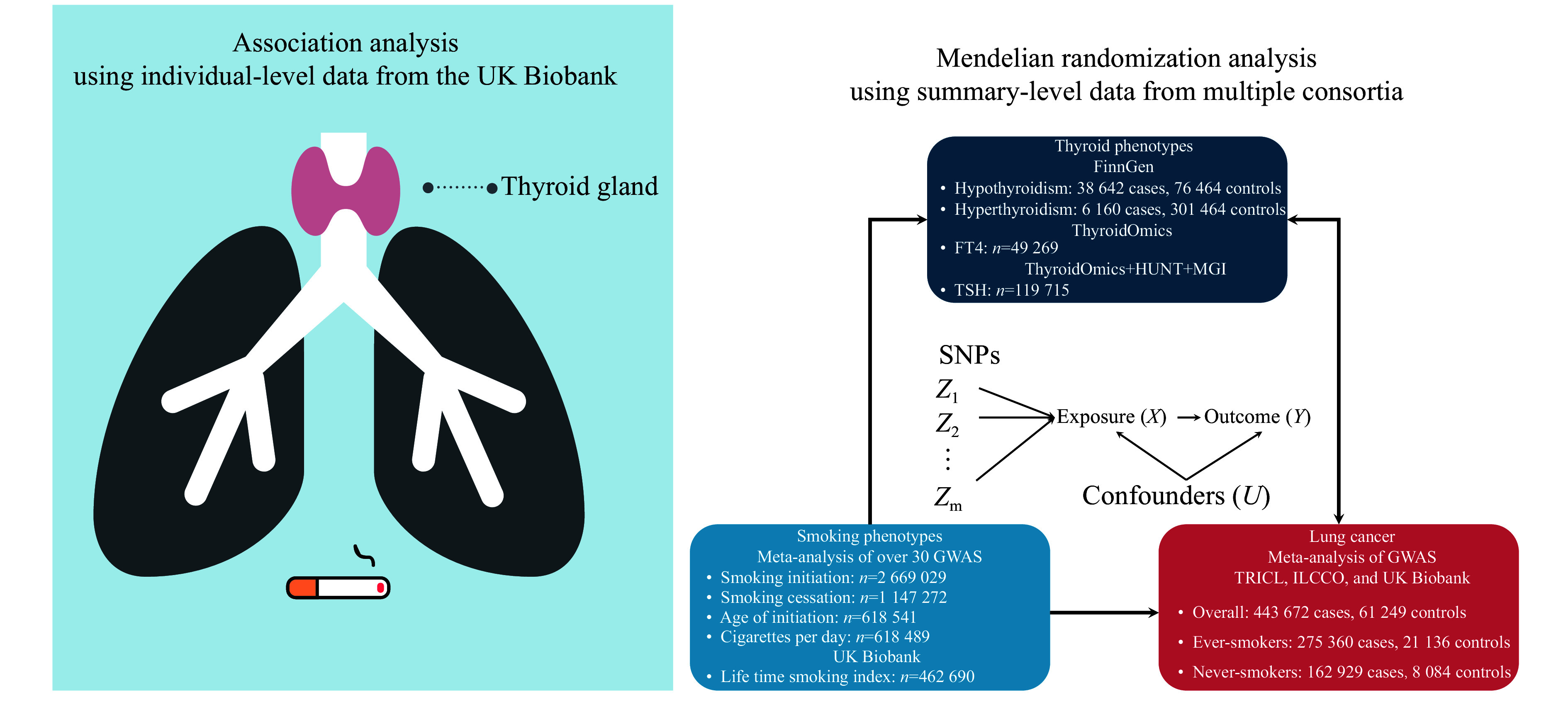
The workflow diagram of the current study. The analytic strategy consisted of two parts. First, individual-level data from the UK Biobank, a large prospective cohort, were used to explore the associations among smoking, thyroid dysfunction, and lung cancer. Second, genetic evidence for the causality of thyroid dysfunction in lung cancer was derived from bidirectional Mendelian randomization using publicly available summary-level data. Abbreviations: FT4, free thyroxine; GWAS, genome-wide association study; HUNT, the Trøndelag Health Study; ILCCO, International Lung Cancer Consortium; MGI, Michigan Genomics Initiative; SNP, single-nucleotide polymorphism; TRICL, Transdisciplinary Research in Cancer of the Lung; TSH, thyroid-stimulating hormone.

The analytic strategy consisted of two parts. First, we used individual-level data from the large prospective UKB cohort to explore the associations among smoking, thyroid dysfunction, and lung cancer risk. Second, we assessed the causal effect between thyroid-related phenotypes and lung cancer risk by performing a bidirectional two-sample MR analysis. Additionally, the stratified MR analysis was performed based on smoking-behavior-specific GWAS of lung cancer risk. Furthermore, we conducted MR-based mediation analysis to determine the mediating role of thyroid-related phenotypes in the association between smoking behavior and lung cancer risk (***Fig. 1***).

#### Individual-level association analysis

Because FT4 and TSH were not available in the UKB, the Cox proportional hazards regression models were used to assess the associations between thyroid dysfunction and lung cancer risk, adjusted for age, sex, and BMI. We also applied a multivariable logistic regression model to investigate the associations among smoking status, thyroid dysfunction, and lung cancer risk.

#### Two-sample MR analysis based on meta-GWAS summary-level data

We used the two-sample MR analysis to assess the causal relationships between thyroid-related phenotypes (thyroid dysfunction for hyperthyroidism and hypothyroidism as well as thyroid-related phenotypes for FT4 and TSH) and lung cancer risk. SNPs associated with each exposure factor at a genome-wide significance level (*P* < 5 × 10^−8^) were regarded as IVs. Additionally, the approximately independent SNPs with linkage disequilibrium (LD) at *r*^2^ ≤ 0.1 were filtered out using the LD clumping algorithm^[28]^. We harmonized all IVs for each trait to ensure that the effect allele was the same for both exposures and outcomes. Palindromic SNPs and SNPs with incompatible alleles were excluded from the current study. We also calculated the *F*-statistic to evaluate the strength of the IVs, and SNPs with an *F*-statistic < 10, which were considered a weak instrument effect, were deleted. Then we removed the SNPs that were significantly associated with both thyroid phenotypes and lung cancer to avoid pleiotropy.

In the primary analysis, the causal effects of thyroid-related phenotypes on lung cancer risk were estimated using the random-effects inverse-variance weighted (MR-IVW) method^[29]^. In sensitivity analysis, we performed a series of different MR methods, including Egger regression (MR-Egger), maximum likelihood (MR-Maxlik)^[30]^, robust adjusted profile score (MR-RAPS), radial (MR-Radial)^[31]^, generalized summary-data-based MR (GSMR)^[32]^, and MR pleiotropy residual sum and outlier (MR-PRESSO)^[33]^, to verify the robustness of results. We also generated scatter plots of the SNP effect of each exposure factor against the effect of outcomes. Causal estimates using each instrument were displayed visually by funnel plots to assess potential asymmetries. Moreover, we carried out a leave-one-out analysis, which sequentially omitted one SNP at a time to further investigate whether the causal estimate was driven or biased by a single SNP. An additional sensitivity analysis was performed using the MRlap R-package to address overfitting bias caused by sample overlap^[34]^. Additionally, a reverse MR analysis was used to clarify the direction of the causal relationship between thyroid-related phenotypes and lung cancer risk. The stratified MR analysis was performed to explore the specific population and specific lung cancer histology.

Furthermore, the MR-based mediation analysis was performed using the product of coefficients method in a two-step MR framework to investigate the proportion of the effect of smoking phenotypes on lung cancer risk mediated through thyroid-related phenotypes^[35]^.

Additionally, we developed a genetic risk score (GRS) by combining the effects of candidate SNPs that were associated with thyroid dysfunction from the GWAS summary-level data in the FinnGen project, and reproduced the GRS using the GWAS individual-level data in the UKB^[36]^. We constructed a weighted GRS to integrate the genetic effects of candidate SNPs on the exposure of interest for available individual-level genotyping data. Then, we evaluated the association between the GRS of thyroid dysfunction and lung cancer risk through logistic regression and the Cox proportional hazards regression model.

Normally distributed continuous variables were presented as mean ± standard deviation (SD), while non-normally distributed continuous variables were described using the median and interquartile range, and categorical variables were reported as counts and percentages. The correction of type Ⅰ error for multiple testing was performed by the false discovery rate (FDR) method with the "p.adjust" package in R software^[37]^. All analyses were performed using R software (version 3.6.3; R Foundation for Statistical Computing, Vienna, Austria).

## Results

### Association analysis based on individual-level data

In the UKB cohort, hypothyroidism was significantly associated with an increased incidence of lung cancer. The Cox proportional hazards model revealed a hazard ratio (HR) of 1.18 (95% confidence interval [CI] = 1.07–1.30, *P* = 0.001), while the logistic regression model showed an odds ratio (OR) of 1.17 (95% CI = 1.06–1.29, *P* = 0.002) in the overall population (***Table 1***). Similarly, hyperthyroidism was significantly associated with an increased incidence of lung cancer in the overall population. The Cox model yielded an HR of 1.64 (95% CI = 1.38–1.96, *P* = 3.96 × 10^−8^), and the logistic regression model showed an OR of 1.64 (95% CI = 1.37–1.96, *P* = 7.48 × 10^−8^). For ever-smokers, the observational study showed results consistent with those in the overall population.

**Table 1:** Results of the association analysis of smoking, thyroid dysfunction, and lung cancer risk in the UK Biobank cohort^a^

Exposure	Outcome	Exposure proportion in population [*n* (%)]	Population	Cox proportional hazards regression			Logistic regression	
				HR (95% CI)	*P*		OR (95% CI)	*P*
Hypothyroidism	Lung cancer	36580 (7.31)	Overall^b^	1.18 (1.07, 1.30)	0.001		1.17 (1.06, 1.29)	0.002
		21499 (7.22)	Ever-smokers^c,d^	1.18 (1.06, 1.31)	0.002		1.17 (1.05, 1.30)	0.004
		14859 (7.41)	Never-smokers^c,d^	1.21 (0.93, 1.57)	0.166		1.20 (0.92, 1.56)	0.181
Hyperthyroidism	Lung cancer	7189 (1.44)	Overall^b^	1.64 (1.38, 1.96)	3.96×10^−8^		1.64 (1.37, 1.96)	7.48×10^−8^
		4374 (1.47)	Ever-smokers^c,d^	1.18 (1.06, 1.31)	2.46×10^−3^		1.56 (1.28, 1.90)	9.93×10^−6^
		2768 (1.38)	Never-smokers^c,d^	2.22 (1.44, 3.44)	3.38×10^−4^		2.23 (1.44, 3.47)	3.32×10^−4^
Smoke	Hypothyroidism	297570 (59.43)	Overall^c^	–	–		1.07 (1.04, 1.09)	5.92×10^−9^
Smoke	Hyperthyroidism	297570 (59.43)	Overall^c^	–	–		1.16 (1.11, 1.22)	1.32×10^−9^
^a^The data of FT4 and TSH were not available in the UK Biobank.^b^Model adjusted for smoking status, age, sex, and BMI.^c^Model adjusted for age, sex, and BMI.^d^Some participants did not provide smoking status.Abbreviations: CI, confidence interval; HR, hazard ratio; OR, odds ratio.								

### Causal associations between thyroid-related phenotypes and lung cancer risk

**Figure 2:**
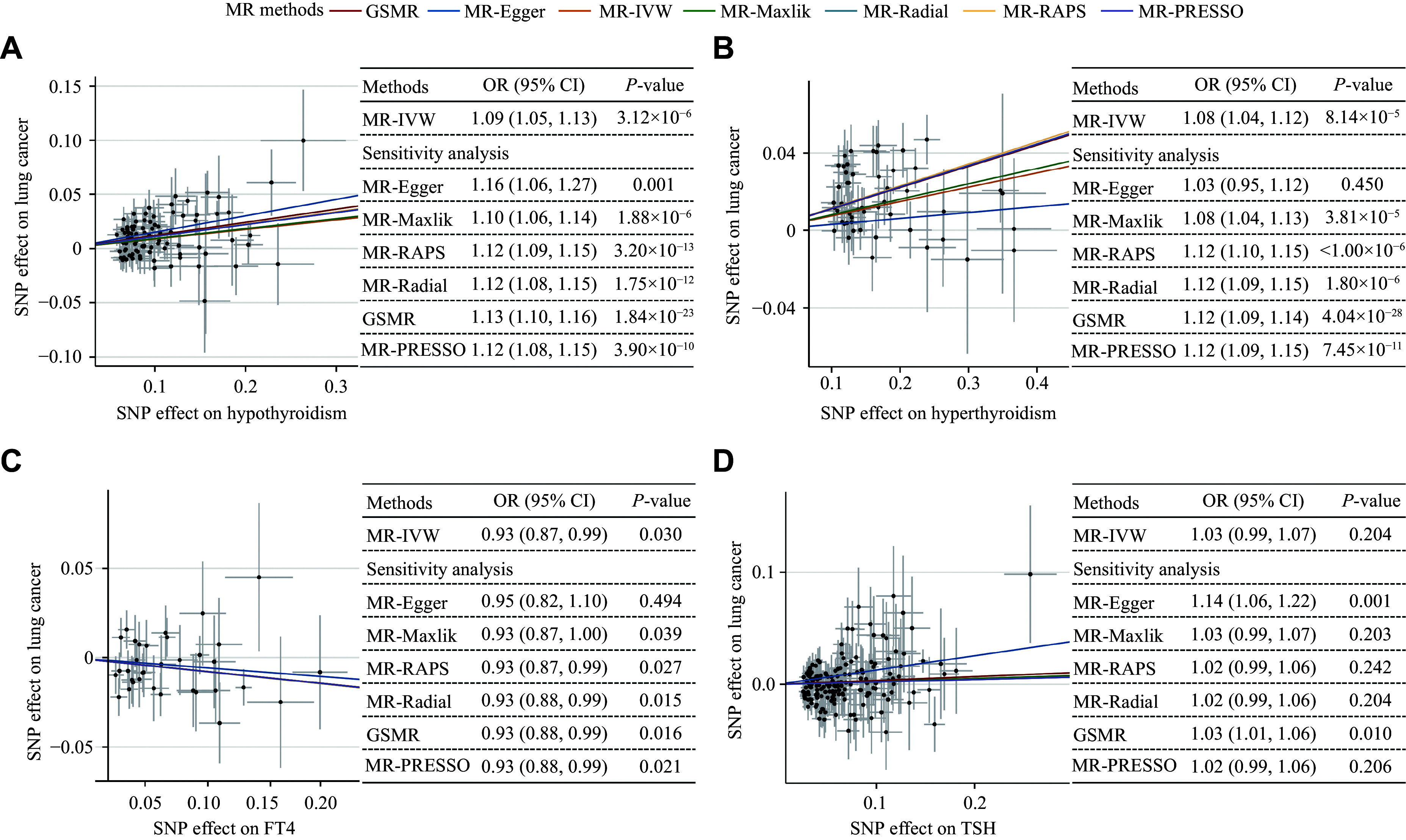
Scatter plots showing the associations between thyroid function-related phenotypes and lung cancer in the overall population. Each cross represents an instrumental variable. Error bars indicate 95% CIs. The lines with different colors were estimated by different MR methods. The slope of each line represents the estimated association of thyroid function-related phenotypes and lung cancer risk. A: Causal association between hypothyroidism and lung cancer using different MR methods. B: Causal association between hyperthyroidism and lung cancer using different MR methods. C: Causal association between FT4 and lung cancer using different MR methods. D: Causal association between TSH and lung cancer using different MR methods. Abbreviations: CI, confidence interval; FT4, free thyroxine; GSMR, generalized summary-data-based Mendelian randomization; MR, Mendelian randomization; MR-Egger, Mendelian randomization with Egger regression; MR-IVW, inverse-variance weighted two-sample Mendelian randomization; MR-Maxlik, Mendelian randomization using maximum-likelihood method; MR-PRESSO, Mendelian randomization pleiotropy residual sum and outlier; MR-Radial, Mendelian randomization with radial regression; MR-RAPS, Mendelian randomization with robust adjusted profile score; OR, odds ratio; SNP, single-nucleotide polymorphism; TSH, thyroid-stimulating hormone.

To determine the causal associations between thyroid-related phenotypes and lung cancer risk, we performed a two-sample MR analysis. After excluding SNPs according to the criteria of IVs, we obtained a final set of 89 SNPs for hypothyroidism, 51 SNPs for hyperthyroidism, 34 SNPs for FT4, and 173 SNPs for TSH. The instrumental strengths (*F*-statistics) for all included SNPs were larger than 10, indicating little weak instrumental bias (***Supplementary Tables 4–7***, available online). As shown in ***Fig. 2***, there was strong evidence that both hypothyroidism and hyperthyroidism were causal risk factors for lung cancer. In the primary analysis, we applied IVW and obtained OR_inverse variance weighted [IVW]_ of 1.09 (95% CI = 1.05–1.13, *P* = 3.12 × 10^−6^) and 1.08 (95% CI = 1.04–1.12, *P* = 8.14 × 10^−5^) for hypothyroidism and hyperthyroidism, respectively. Additionally, we found that a per SD increase in FT4 concentration was negatively associated with lung cancer risk (OR_IVW_ = 0.93, 95% CI = 0.87–0.99, *P* = 0.030). Meanwhile, there was no evidence supporting the causal effect of TSH on lung cancer risk. Furthermore, a series of other MR methods yielded consistent results, indicating the robustness of the causal effects between the three thyroid-related phenotypes and lung cancer risk (***Fig. 2***).

In the sensitivity analysis, the estimated causal effects of each hypothyroidism- and hyperthyroidism-associated SNP were symmetrically distributed in the funnel plot (***Supplementary Figs. 1*** and ***2***, available online). The leave-one-out analysis did not identify any variants with an inflationary effect on the causal estimations (***Supplementary Figs. 3***–***6***, available online). The findings from the MRlap analysis consistently supported causal associations between thyroid-related phenotypes and the risk of lung cancer, and there was no evidence of directional pleiotropy, as indicated by the MR-Egger intercept value. Additionally, positive effects were observed for hypothyroidism and the risk of squamous cell carcinoma (OR_IVW_ = 1.23, 95% CI = 1.15–1.31, *P* = 6.84 × 10^−10^) and small cell carcinoma (OR_IVW_ = 1.12, 95% CI = 1.03–1.20, *P* = 4.70 × 10^−3^). Consistent with the overall lung cancer results, we also observed the effect of hyperthyroidism on squamous cell carcinoma (OR_IVW_ = 1.16, 95% CI = 1.10–1.23, *P* = 6.08 × 10^−8^) and the effect of FT4 on small cell carcinoma (OR_IVW_ = 0.81, 95% CI = 0.66–0.99, *P* = 0.04). The MR analysis showed that TSH had an estimated effect of increasing the risk of adenocarcinoma (OR_IVW_ = 1.12, 95% CI = 1.06–1.20, *P* = 2.26 × 10^−4^) (***Supplementary Fig. 7***, available online).

Moreover, reverse MR analysis showed some evidence that the increased risk of lung cancer was also causally associated with a higher risk of hyperthyroidism (OR_IVW_ = 1.11, 95% CI = 1.00–1.22, *P* = 0.040), a higher level of FT4 (*β*_IVW_ = 0.04, 95% CI = 0.01–0.08, *P* = 0.025), and a lower level of TSH (*β*_IVW_ = −0.04, 95% CI = −0.06–−0.01, *P* = 0.003) (***Supplementary Fig. 8A***–***8C***, available online). There was no clear evidence of a causal association between lung cancer risk and hypothyroidism (***Supplementary Fig. 8D***, available online).

### Causal associations between smoking phenotypes and thyroid-related phenotypes

**Figure 3:**
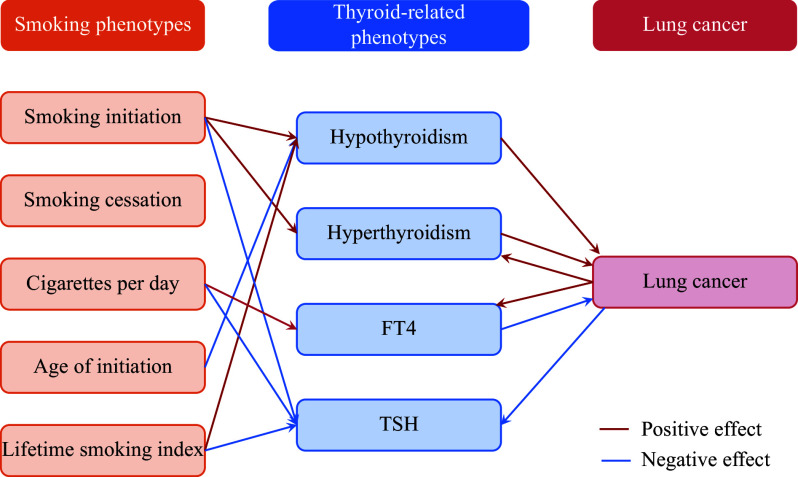
The causal graph of smoking phenotypes, thyroid-related phenotypes, and lung cancer. Significant causal effects are presented as arrows. Red indicates a positive effect, and blue indicates a negative effect. Abbreviations: FT4, free thyroxine; TSH, thyroid-stimulating hormone.

After applying an FDR correction for multiple comparisons, the MR analysis indicated potential causal effects of smoking phenotypes on thyroid dysfunction. Smoking initiation was associated with a higher risk of both hypothyroidism (OR_IVW_ = 1.19, 95% CI = 1.07–1.33, *q-FDR* = 0.004) and hyperthyroidism (OR_IVW_ = 1.56, 95% CI = 1.30–1.88, *q-FDR* = 3.62 × 10^−5^), as well as lower levels of TSH (*β*_IVW_ = −0.10, 95% CI = −0.15–−0.05, *q-FDR* = 4.21 × 10^−4^). Regarding cigarettes per day, the effects were reversed for FT4 (*β*_IVW_ = 0.13, 95% CI = 0.04–0.21, *q-FDR* = 0.016) and TSH (*β*_IVW_ = −0.12, 95% CI = −0.19–−0.06, *q-FDR* = 0.001). An increase in the age of initiation was causally associated with a lower risk of hypothyroidism (OR_IVW_ = 0.31, 95% CI = 0.15–0.63, *q-FDR* = 0.004). Additionally, a one-SD increase in the lifetime smoking index was causally associated with an increased risk of hypothyroidism (OR_IVW_ = 1.73, 95% CI = 1.29–2.34, *q-FDR* = 0.001) as well as a decreased level of TSH (*β*_IVW_ = −0.11, 95% CI = −0.18–−0.03, *q-FDR* = 0.022) (***Supplementary Fig. 9*** [available online] and ***Fig. 3***).

### Smoking behavior modified the causal effect of thyroid-related phenotypes on lung cancer risk

**Figure 4:**
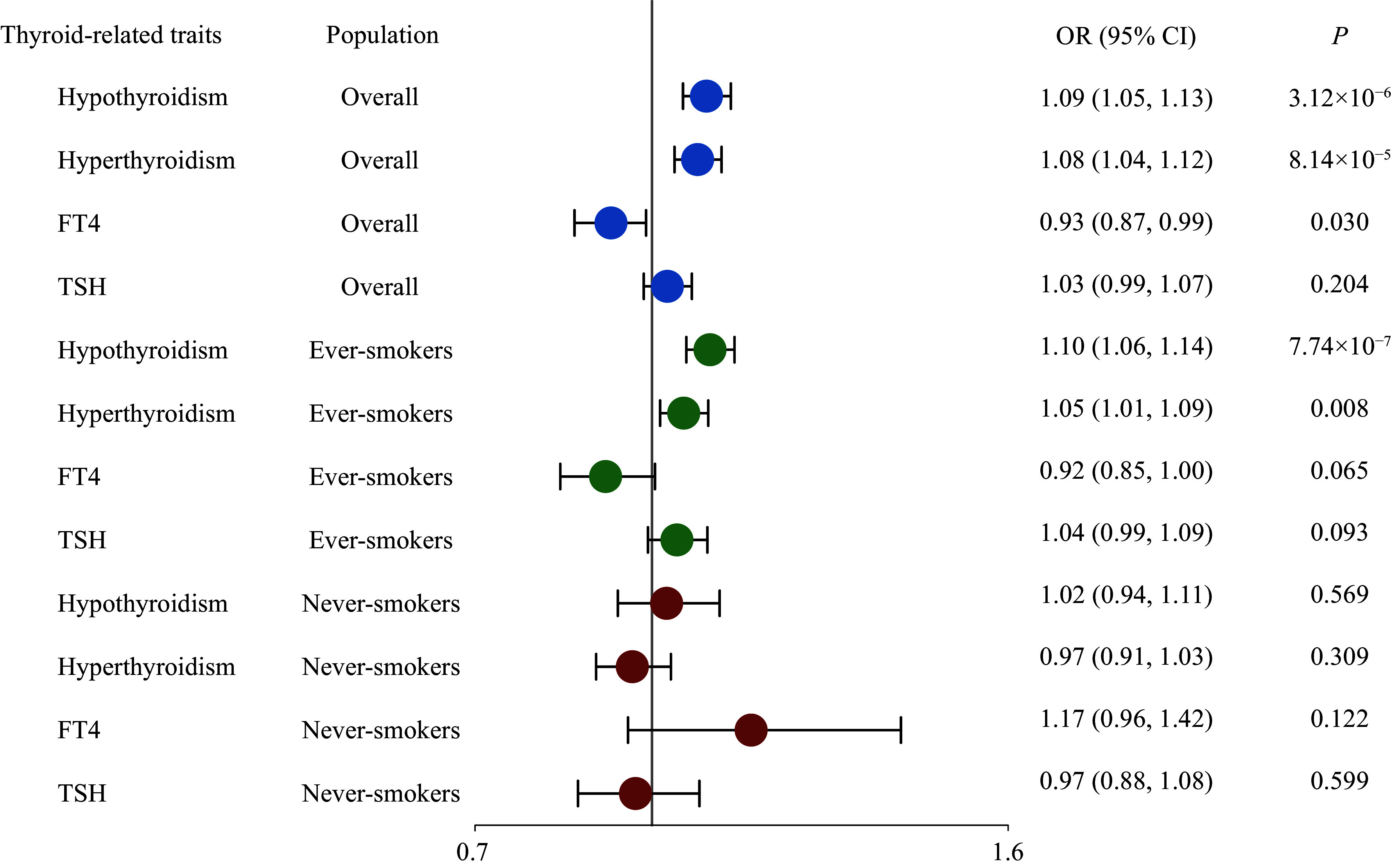
Forest plots for the results of stratified MR analysis. The red circle indicates the overall population, the blue circle indicates ever-smokers, and the green circle indicates never-smokers. Abbreviations: CI, confidence interval; FT4, free thyroxine; OR, odds ratio; TSH, thyroid-stimulating hormone.

Further, a stratified MR analysis was performed to explore the causal effects of thyroid-related phenotypes on lung cancer risk. Both hypothyroidism and hyperthyroidism were causally associated with lung cancer only among ever-smokers, while no evidence was found in non-smokers (***Fig. 4***). Moreover, the direction of the causal effect of each phenotype remained consistent with that observed in the overall population (OR_IVW_ = 1.10, 95% CI = 1.06–1.14, *P* = 7.74 × 10^−7^ for hypothyroidism; OR_IVW_ = 1.05, 95% CI = 1.01–1.09, *P* = 0.008 for hyperthyroidism). A series of sensitivity analyses revealed a robust causal association between thyroid dysfunction and lung cancer risk among the ever-smoking population (***Supplementary Figs. 10***–***16***, available online).

### Thyroid-related phenotypes mediated the causal effects of smoking phenotypes on lung cancer

**Figure 5:**
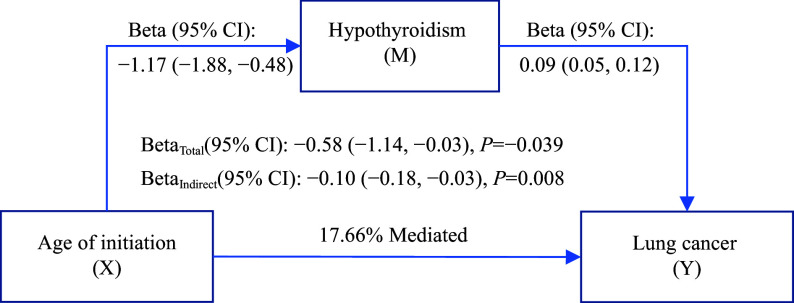
Mediation analysis for the association between thyroid-related phenotypes, smoking phenotypes, and lung cancer risk. The *β* (beta) values and 95% CIs represent the indirect effects of smoking phenotypes on lung cancer risk mediated through thyroid-related phenotypes. Abbreviations: CI, confidence interval; M, mediator; X, exposure; Y, outcome.

The mediating effects of thyroid-related phenotypes on the association between smoking phenotypes and lung cancer were estimated by a two-step MR analysis. The causal associations of smoking phenotypes and lung cancer with thyroid-related phenotypes were examined using two-sample MR analysis (***Fig. 3***). The proportion of the total effect of the age of smoking initiation on lung cancer risk mediated by hypothyroidism was 17.66% (***Fig. 5***). Additionally, the proportion of the total effect of smoking initiation on lung cancer risk mediated by hypothyroidism and hyperthyroidism was estimated to be 2.02% (***Supplementary Fig. 17A***, available online) and 4.31% (***Supplementary Fig. 17B***, available online), respectively. Hypothyroidism mediated 2.49% of the total effect of the lifetime smoking index on lung cancer risk (***Supplementary Fig. 17C***, available online).

Subsequently, similar evidence was found supporting the associations of genetically predicted hypothyroidism and hyperthyroidism with the risk of lung cancer in the overall population of the UKB cohort study (***Supplementary Table 8***, available online).

## Discussion

To the best of our knowledge, the current study is the first attempt to explore the causal relationship among smoking, thyroid-related phenotypes, and lung cancer using both observational and genetic evidence. Our study revealed robust causal associations between thyroid-related phenotypes (hypothyroidism, hyperthyroidism, and TSH) and lung cancer. Through stratified analysis by smoking status, we observed that these causal associations between thyroid dysfunction and lung cancer appeared exclusively among smokers. Additionally, there was sufficient evidence for reverse causal associations between thyroid-related phenotypes and lung cancer, except in the case of hypothyroidism.

Observational studies have indicated that both thyroid hormone levels and thyroid disorders are associated with overall cancer risk, including breast cancer, prostate cancer, and colorectal cancer^[38–41]^. While few previous studies have addressed the association between thyroid-related phenotypes and lung cancer risk, our investigation contributes to the literature by providing interesting and promising results. Notably, our study highlights the causal role of thyroid dysfunction in lung cancer, especially among smokers, which is consistent with previous epidemiological findings^[10,39]^. The underlying mechanism may involve the effect of tobacco smoking on thyroid gland function^[42]^. Tobacco contains cyanide, which is converted into the chemical thiocyanate when smoked. Thiocyanate is known to interfere with thyroid-related phenotypes by inhibiting iodine uptake into the thyroid gland and reducing thyroid hormone production^[43–45]^. This suggests that smoking may lead to disruptions in thyroid hormone production, resulting in thyroid dysfunction and an increased risk of lung cancer.

As for the association between thyroid hormone levels and lung cancer, the current results are inconsistent with those of previous studies. In the Rotterdam study^[10]^, higher FT4 levels were associated with an increased risk of lung cancer, while no significant association was found between TSH levels and lung cancer risk. In contrast, Chan *et al*^[12]^ found that lower TSH and higher FT4 levels predicted the incidence of prostate cancer but not lung cancer in Western Australia. Unsurprisingly, the findings of these prospective cohort studies were also conflicting. Possible explanations for these discrepancies include unmeasured confounding factors or reverse causality. Although efforts have been made to adjust for obvious confounders, traditional statistical methods struggle to fully account for these factors in observational studies. Another plausible reason could be insufficient power because of the small sample size of lung cancer in each study (ranging from 41 to 201).

Inconsistencies also exist between the current study and previous MR results^[46]^, which may be attributed to several factors, including differences in phenotype definitions, GWAS sample size, and the selection of instrumental variables. First, the phenotypes used in Wang's study were "hypothyroidism, strict autoimmune" and "autoimmune hyperthyroidism", whereas the current study included "hypothyroidism (congenital or acquired)", "autoimmune hyperthyroidism", and "thyrotoxicosis". Second, the summary-level data for lung cancer in the current study were obtained from a meta-analysis of populations in the UKB, TRICL, and ILLCO, including 34056 lung cancer cases and 470856 controls. Compared with Wang's study, the current study involved a larger and more diverse sample size, providing greater statistical power. Third, Wang's study selected a stricter threshold (0.001) for *r*^2^ in the LD pruning process, which led to fewer SNPs being selected as valid instruments and reduced analytic power. Additionally, the current study incorporated data from the UKB^[46]^.

In general, we demonstrated that abnormal thyroid hormone levels were associated with the development of lung cancer, which aligns with the role of thyroid hormone in cancer pathogenesis. Several studies have reported that immune-related thyroid dysfunction is associated with the response to anti-PD-1 therapy among patients with non-small cell lung cancer^[47]^, in which Luo *et al*^[47]^ identified that genetic differences in immunity might contribute to toxicity and outcomes in immune checkpoint inhibitor therapy. Our bidirectional MR results regarding the association between thyroid dysfunction and lung cancer provide insights into the associations among underlying autoimmunity, immune-related thyroid dysfunction, and immunotherapy outcomes.

Notably, our results indicate that hereditary hypothyroidism increases the risk of lung cancer, especially among smokers. Some studies have elucidated the underlying mechanisms by which hypothyroidism contributes to the development of lung cancer. Evidence from animal experiments^[48]^ and clinical studies^[49]^ suggests that hypothyroidism affects the hypothalamic-pituitary-gonadal axis and is associated with mitochondrial dysfunction, leading to an increased production of reactive oxygen species (ROS)^[50]^. The tumor-promoting effect of ROS in lung cancer has been well demonstrated. Accumulating evidence^[51–53]^ has also suggested that ROS plays a major role in the initiation, promotion, and progression of cancer by regulating signal molecules involved in cell proliferation, angiogenesis, and the alteration of the migration and invasion programs^[53–54]^. Another potential mechanism may involve the derivative of L-thyroxine tetrac^[55]^. Tetrac is a minor product in normal thyroid physiology, which inhibits tumor growth by blocking the binding of thyroid hormones to the plasma membrane receptor integrin αvβ3^[56–58]^. Increasing evidence suggests that tetrac is involved in anti-angiogenic and anti-tumor activities, including the inhibition of cancer cell proliferation^[59–60]^, the enhancement of cancer cell apoptosis, and the disruption of multiple angiogenic pathways. Furthermore, tetrac has been shown to effectively inhibit the growth of non-small cell lung cancer *in vitro* as well as in chick chorioallantoic membrane assay and murine xenograft models^[61]^.

The current study has several strengths. First, the MR analysis was used to identify the association between thyroid dysfunction and lung cancer risk, thus avoiding potential false associations and reverse causality, which are common in observational studies. Second, we applied a series of MR methods and sensitivity analyses to verify the robust causal effect of thyroid dysfunction on lung cancer and conducted an independent validation using individual-level data from a large prospective cohort. Third, the current study included the largest sample of lung cancer cases and thyroid function-related phenotypes to date, ensuring sufficient power to infer causality. Fourth, we also performed a bidirectional MR analysis to clarify the direction of the association between thyroid dysfunction and lung cancer. Finally, we found that the effect of smoking on lung cancer risk might be mediated through thyroid-related phenotypes.

However, we acknowledge that the current study has some limitations. First, our study is limited to individuals of European ancestry; thus, the findings should be extrapolated to other populations with caution. Second, because of the lack of thyroid hormone level data in the UKB, we were unable to explore the dose-response relationship between TSH and FT4 levels and lung cancer risk. Third, further biological experiments are warranted to explore the causal mechanism underlying the association between thyroid-related phenotypes and lung carcinogenesis.

In conclusion, both observational and causal evidence support the effect of thyroid dysfunction on lung cancer, especially in smokers. Specifically, both hypothyroidism and hyperthyroidism were associated with an increased risk of lung cancer. The findings of the current study may draw attention to the role of thyroid dysfunction in lung carcinogenesis and provide some insights into the biological mechanisms underlying lung cancer prevention and clinical practice. Whether the effects of thyroid dysfunction and hormone levels are meaningful as potential intervention targets requires further investigation to unravel the molecular pathways involved.

## SUPPLEMENTARY DATA

Supplementary data to this article can be found online.

## References

[1] (2023). Cancer statistics, 2023. CA Cancer J Clin.

[2] (2017). Early and locally advanced non-small-cell lung cancer (NSCLC): ESMO Clinical Practice Guidelines for diagnosis, treatment and follow-up. Ann Oncol.

[3] (2017). Lung cancer: Current therapies and new targeted treatments. Lancet.

[4] (2019). Five-year overall survival for patients with advanced non-small-cell lung cancer treated with pembrolizumab: Results from the phase Ⅰ KEYNOTE-001 study. J Clin Oncol.

[5] (2019). Thyroid hormones and cancer: A comprehensive review of preclinical and clinical studies. Front Endocrinol (Lausanne).

[6] (2013). Molecular functions of thyroid hormones and their clinical significance in liver-related diseases. Biomed Res Int.

[7] (2019). Molecular functions of thyroid hormone signaling in regulation of cancer progression and anti-apoptosis. Int J Mol Sci.

[8] (2017). Hyperthyroidism, hypothyroidism, and cause-specific mortality in a large cohort of women. Thyroid.

[9] (2014). Thyroid-stimulating hormone, thyroglobulin, and thyroid hormones and risk of differentiated thyroid carcinoma: The EPIC study. J Natl Cancer Inst.

[10] (2016). Thyroid function and cancer risk: The Rotterdam study. J Clin Endocrinol Metab.

[11] (2020). Thyroid dysfunction and cancer incidence: A systematic review and meta-analysis. Endocr Relat Cancer.

[12] (2017). Lower TSH and higher free thyroxine predict incidence of prostate but not breast, colorectal or lung cancer. Eur J Endocrinol.

[13] (2006). Serum TSH levels in smokers and non-smokers. The 5th Tromsø study. Exp Clin Endocrinol Diabetes.

[14] (1997). Cigarette smoking and thyroid hormone levels in males. Int J Epidemiol.

[15] (1993). Smoking and risk of Graves' disease. JAMA.

[16] (2020). Cigarette smoking is associated with higher thyroid hormone and lower TSH levels: The PREVEND study. Endocrine.

[17] (2016). Mendelian randomization as an approach to assess causality using observational data. J Am Soc Nephrol.

[18] (2018). Reading Mendelian randomisation studies: A guide, glossary, and checklist for clinicians. BMJ.

[19] (2017). Nature as a trialist?: Deconstructing the analogy between Mendelian randomization and randomized trials. Epidemiology.

[20] (2015). UK Biobank: An open access resource for identifying the causes of a wide range of complex diseases of middle and old age. PLoS Med.

[21] (2009). Cohorts for Heart and Aging Research in Genomic Epidemiology (CHARGE) consortium: Design of prospective meta-analyses of genome-wide association studies from 5 cohorts. Circ: Cardiovasc Genet.

[22] (2023). FinnGen provides genetic insights from a well-phenotyped isolated population. Nature.

[23] (2018). Genome-wide analyses identify a role for SLC17A4 and AADAT in thyroid hormone regulation. Nat Commun.

[24] (2020). GWAS of thyroid stimulating hormone highlights pleiotropic effects and inverse association with thyroid cancer. Nat Commun.

[25] (2022). Genetic diversity fuels gene discovery for tobacco and alcohol use. Nature.

[26] (2020). Evidence for causal effects of lifetime smoking on risk for depression and schizophrenia: A Mendelian randomisation study. Psychol Med.

[27] (2017). Large-scale association analysis identifies new lung cancer susceptibility loci and heterogeneity in genetic susceptibility across histological subtypes. Nat Genet.

[28] (2015). Second-generation PLINK: Rising to the challenge of larger and richer datasets. GigaScience.

[29] (2013). Mendelian randomization analysis with multiple genetic variants using summarized data. Genet Epidemiol.

[30] (2010). Genetic variants influencing circulating lipid levels and risk of coronary artery disease. Arterioscler Thromb Vasc Biol.

[31] (2018). Improving the visualization, interpretation and analysis of two-sample summary data Mendelian randomization *via* the Radial plot and Radial regression. Int J Epidemiol.

[32] (2018). Causal associations between risk factors and common diseases inferred from GWAS summary data. Nat Commun.

[33] (2018). Detection of widespread horizontal pleiotropy in causal relationships inferred from Mendelian randomization between complex traits and diseases. Nat Genet.

[34] (2023). Bias correction for inverse variance weighting Mendelian randomization. Genet Epidemiol.

[35] (2021). Mendelian randomisation for mediation analysis: Current methods and challenges for implementation. Eur J Epidemiol.

[36] (2022). Association between circulating vitamin E and ten common cancers: Evidence from large-scale Mendelian randomization analysis and a longitudinal cohort study. BMC Med.

[37] (1995). Controlling the false discovery rate: A practical and powerful approach to multiple testing. J R Stat Soc Series B (Methodological).

[38] (2020). Subclinical hypothyroidism and the risk of cancer incidence and cancer mortality: A systematic review. BMC Endocr Disord.

[39] (2009). Thyroid function and cancer risk: A prospective population study. Cancer Epidemiol Biomarkers Prev.

[40] (2012). Mechanisms in endocrinology: Primary HT and risk for breast cancer: a systematic review and meta-analysis. Eur J Endocrinol.

[41] (2019). Association between thyroid disorders and colorectal cancer risk in adult patients in Taiwan. JAMA Netw Open.

[42] (2013). Smoking and thyroid. Clin Endocrinol (Oxf).

[43] (2018). Effect of perchlorate and thiocyanate exposure on thyroid function of pregnant women from South-West England: A cohort study. Thyroid Res.

[44] (2003). Thiocyanate overload and thyroid disease. Biofactors.

[45] (2014). Influence of cigarette smoking on thyroid gland—an update. Endokrynol Pol.

[46] (2023). The causal relationship between thyroid function, autoimune thyroid dysfunction and lung cancer: A mendelian randomization study. BMC Pulm Med.

[47] (2021). Immunotherapy-mediated thyroid dysfunction: Genetic risk and impact on outcomes with PD-1 blockade in non-small cell lung cancer. Clin Cancer Res.

[48] (2021). Pathological changes and oxidative stress of the HPG axis in hypothyroid rat. J Mol Endocrinol.

[49] (2008). Oxidative stress and protein glycation in primary hypothyroidism. Male/female difference. Clin Exp Med.

[50] (2021). Hypothyroidism induces oxidative stress and DNA damage in breast. Endocr Relat Cancer.

[51] (2008). Inflammation and lung cancer: Roles of reactive oxygen/nitrogen species. J Toxicol Environ Health B Crit Rev.

[52] (2017). The two faces of reactive oxygen species in cancer. Annu Rev Cancer Biol.

[53] (2009). Benzo[a]pyrene promotes proliferation of human lung cancer cells by accelerating the epidermal growth factor receptor signaling pathway. Cancer Lett.

[54] (2010). Mitochondrial metabolism and ROS generation are essential for Kras-mediated tumorigenicity. Proc Natl Acad Sci U S A.

[55] (2005). Alternate pathways of thyroid hormone metabolism. Thyroid.

[56] (2010). Molecular aspects of thyroid hormone actions. Endocr Rev.

[57] (2008). Mechanisms of nongenomic actions of thyroid hormone. Front Neuroendocrinol.

[58] (2015). Thyroid hormones and tetrac: New regulators of tumour stroma formation *via* integrin αvβ3. Endocr Relat Cancer.

[59] (2019). Tetrac as an anti-angiogenic agent in cancer. Endocr Relat Cancer.

[60] (2021). Nongenomic actions of thyroid hormone: The integrin component. Physiol Rev.

[61] (2012). Tetraiodothyroacetic acid and its nanoformulation inhibit thyroid hormone stimulation of non-small cell lung cancer cells *in vitro* and its growth in xenografts. Lung Cancer.

