# Supplementary material for: Bidirectional Mendelian randomization and mediation analysis of million-scale data reveal causal relationships between thyroid-related phenotypes, smoking, and lung cancer
Source: J Biomed Res. 2025 Mar 10;39(5):441–51. doi: 10.7555/JBR.38.20240421 (PMC12481675; doi:10.7555/JBR.38.20240421)
Supplement: Supplementary file 1 — Supplementary data to this article can be found online. [file jbr-39-5-441-Supplementary.pdf]

Available online at www.jbr-pub.org.cn

Open Access at PubMed Central

Journal of Biomedical Research, 2025 39(5): 1–23

JBR

Supplementary Data

Bidirectional Mendelian randomization and mediation analysis of million-scale data reveal causal relationships between thyroid-related phenotypes, smoking, and lung cancer

Xiang Wang[1,2,3,△], Xuan Wang[1,△], Mengsheng Zhao[1,△], Lijuan Lin[1,△], Yi Li[4], Ning Xie[1], Yanru Wang[1], Aoxuan Wang[1], Xiaowen Xu[1], Can Ju[1], Qiuyuan Chen[1], Jiajin Chen[1], Ruili Hou[1], Zhongwen Zhang[1], David C. Christiani[5,6], Feng Chen[1,2,3,✉], Yongyue Wei[1,7,✉], Ruyang Zhang[1,2,8,✉]

[1]Department of Biostatistics, Center for Global Health, School of Public Health, Nanjing Medical University, Nanjing, Jiangsu 211166, China;
[2]China International Cooperation Center for Environment and Human Health, Nanjing Medical University, Nanjing, Jiangsu 211166, China;
[3]Jiangsu Key Lab of Cancer Biomarkers, Prevention and Treatment, Cancer Center, Collaborative Innovation Center for Cancer Personalized Medicine, Nanjing Medical University, Nanjing, Jiangsu 211166, China;
[4]Department of Biostatistics, University of Michigan, Ann Arbor, MI 48109, USA;
[5]Pulmonary and Critical Care Division, Department of Medicine, Massachusetts General Hospital and Harvard Medical School, Boston, MA 02114, USA;
[6]Department of Environmental Health, Harvard T.H. Chan School of Public Health, Boston, MA 02115, USA;
[7]Center for Public Health and Epidemic Preparedness & Response, Peking University, Beijing 100191, China;
[8]Changzhou Medical Center, Nanjing Medical University, Changzhou, Jiangsu 213164, China.

Supplementary Table 1 **Demographic and clinical descriptions of lung cancer cases and controls in the UK Biobank[a]**

Characteristics	Case (*n*=5 119)	Control (*n*=495 595)
Age (years)	61.58±8.10	56.46±5.95
BMI	27.41±4.78	27.43±4.80
Sex (%)		
Female	2 504 (48.92)	270 171 (54.51)
Male	2 615 (51.08)	225 424 (45.49)
Smoking status (%)[b]		
Never	597 (11.78)	199 814 (40.54)
Ever[c]	4 469 (88.22)	293 101 (59.46)
Smoking pack years	35.62 (22.25 49.00)	18.50 (9.50 31.50)
Hypothyroidism (%)		
No	4 640 (90.64)	459 494 (92.72)
Yes	479 (9.36)	36 101 (7.28)
Hyperthyroidism (%)		
No	4 988 (97.44)	488 537 (98.58)
Yes	131 (2.56)	7 058 (1.42)

[a]The data of FT4 and TSH were not available in the UK Biobank.
[b]Some participants did not provide smoking status.
[c]Ever-smokers include both current and former smokers.
Abbreviations: BMI, body mass index; FT4, free thyroxine; TSH, thyroid-stimulating hormone.

△These authors contributed equally to this work.

✉Corresponding authors: Yongyue Wei, Center for Public Health and Epidemic Preparedness & Response, Peking University, 38 Xueyuan Road, Haidian District, Beijing 100191, China. E-mail: ywei@pku.edu.cn; Feng Chen and Ruyang Zhang, School of Public Health, Nanjing Medical University, 101 Longmian Avenue, Nanjing, Jiangsu 211166, China. E-mails: fengchen@njmu.edu.cn (Chen) and zhangruyang@njmu.edu.cn (Zhang).

Received: 28 November 2024; Revised: 16 February 2025; Accepted: 18 February 2025; Published online: 10 March 2025

CLC number: R734.2, Document code: A

The authors reported no conflict of interests.

This is an open access article under the Creative Commons Attribution (CC BY 4.0) license, which permits others to distribute, remix, adapt and build upon this work, for commercial use, provided the original work is properly cited.

© 2025 by Journal of Biomedical Research.

https://doi.org/10.7555/JBR.38.20240421

Supplementary Table 2 **Phenotypic descriptive statistics for traits: smoking initiation, smoking cessation, the initiation age of regular smoking, and cigarettes per day**

Study name	Smoking initiation[a] (N^e=2 669 029)				Smoking cessation[b] (N^e=1 147 272)				Cigarettes per day[c] (N^e=618 489)					Initiation age of regular smoking[d] (N^e=618 541)				
	n	% Ever smoker	MAF> 0.01	0.01≤ MAF> 0.001	*n*	% Current smoker	MAF> 0.01	0.01≤ MAF> 0.001	*n*	Mean	Var	MAF> 0.01	0.01≤ MAF> 0.001	*n*	Mean	Var	MAF> 0.01	0.01≤ MAF> 0.001
23andMe (EUR) males	866 242	43%	2.13	1.14	362 467	23%	1.18	1.04	129 471	2.38[f]	2.76[f]	1.18	1.02	129 200	17.35	18.42	1.11	1.01
Females	997 356	41%	1.93	1.13	396 492	25%	1.30	1.07	162 521	2.30	2.30	1.22	1.03	165 955	16.95	18.80	1.11	1.02
ALSPAC	11 345	42%	1.00	1.01	4 748	65%	1.00	0.99	4 314	3.40	2.50	1.01	1.00	4 691	16.00	7.80	1.00	1.01
ARIC-TOPMed	6 010	60%	1.04	1.09	3 575	41%	1.02	0.99	3 494	2.97	1.17	1.01	0.92	3 485	18.72	23.80	1.00	0.90
BEAGESS	4 293	65%	1.02	1.03	2 805	20%	1.02	1.07	—	—	—	—	—	—	—	—	—	—
BLS	1 238	46%	1.05	1.03	548	48%	1.03	1.00	546	0.70	0.70	1.04	1.02	546	15.70	6.40	1.02	1.05
CADD	1 192	85%	1.01	1.00	1 002	33%	1.01	0.95	523	2.40	1.40	1.01	1.06	775	16.10	0.40	1.01	0.89
COGEND	—	—	—	—	1954	69%	1.02	1.01	1940	2.60	2.00	1.01	0.98	1952	13.70	10.70	0.99	1.01
COPDGene-TOPMed	—	—	—	—	6 520	39%	0.99	0.84	6 520	3.43	0.91	0.99	0.80	6 520	17.02	17.12	0.96	0.87
deCODE	57 097	70%	1.11	1.05	34 820	37%	1.08	1.06	44 505	0.9[g]	0.7[g]	1.14	1.08	40 314	9.5[h]	8.4[h]	1.06	1.01
EGCUT	48 038	42%	1.13	1.07	20 210	67%	1.04	1.03	19 732	2.08	0.67	1.06	1.03	19 511	18.69	1.06	1.02	1.01
eMERGE-Cataracts	3 873	49%	1.01	1.01	1884	17%	1.01	1.01	—	—	—	—	—	—	—	—	—	—
eMERGE-PAD	1 350	74%	1.01	1.13	1 002	52%	1.03	1.18	—	—	—	—	—	—	—	—	—	—
FinnTwin 1	1 006	54%	1.01	1.03	498	76%	1.01	1.05	498	2.20	0.60	1.02	0.99	509	16.10	4.60	1.00	1.03
FinnTwin 2	8 259	48%	1.02	1.07	4 163	55%	1.01	1.02	3 913	14.85	77.90	1.01	1.03	3 913	19.25	23.64	1.01	1.04
Framingham-TOPMed	4 076	55%	1.01	1.00	2 241	59%	1.00	0.98	1 588	2.80	1.33	0.99	1.00	1 399	17.63	9.99	1.00	0.99
Genes for Good	6 151	39%	1.01	1.03	2 413	31%	1.02	1.09	2 209	2.16	0.94	0.99	0.97	2 198	17.98	16.19	0.99	1.02
GERA	—	—	—	—	—	—	—	—	—	—	—	—	—	—	—	—	—	—
Harvard-Affy	6 901	56%	1.01	1.02	3 883	22%	1.01	1.06	3 788	2.82	1.31	1.01	1.00	2 160	19.70	15.21	1.00	1.00
Harvard-Illumina	6 456	50%	1.01	1.03	3 222	22%	1.01	1.06	3 168	2.69	1.24	1.02	1.01	2 390	19.11	11.69	1.01	0.99

Supplementary Table 2 **Phenotypic descriptive statistics for traits: smoking initiation, smoking cessation, the initiation age of regular smoking, and cigarettes per day** (Continued)

Study name	Smoking initiation[a] (N^e=2 669 029)				Smoking cessation[b] (N^e=1 147 272)				Cigarettes per day[c] (N^e=618 489)					Initiation age of regular smoking[d] (N^e=618 541)				
	n	% Ever smoker	MAF> 0.01	0.01≤ MAF> 0.001	*n*	% Current smoker	MAF> 0.01	0.01≤ MAF> 0.001	*n*	Mean	Var	MAF> 0.01	0.01≤ MAF> 0.001	*n*	Mean	Var	MAF> 0.01	0.01≤ MAF> 0.001
Harvard-HumanCore	7 829	44%	1.03	1.03	3 467	22%	1.01	1.05	3 395	2.69	1.28	1.00	1.00	2 464	17.90	14.41	1.01	1.00
Harvard-OmniExpress	7 819	54%	1.01	1.02	4 258	22%	1.02	1.05	4 153	2.73	1.25	1.02	1.01	2 734	19.70	13.69	1.00	1.00
Harvard-OncoArray	9 087	46%	1.03	1.03	4 137	24%	1.01	1.05	4 051	2.62	1.22	1.01	1.01	3 296	18.32	12.65	1.00	1.01
HRS	9 989	57%	1.03	1.02	—	—	—	—	5 306	2.90	1.80	1.00	1.00	5 585	17.70	32.70	1.00	0.99
HUNT	66 716	57%	1.20	1.10	37 964	58%	1.15	1.10	33 705	2.10	0.50	1.05	1.05	35 311	18.80	26.20	1.05	1.05
MCTFR	6 181	45%	1.02	1.05	2 808	67%	1.00	1.02	2 535	2.60	2.30	1.00	1.03	—	—	—	—	—
MESA-TOPMed	1963	56%	0.98	0.99	—	—	—	—	1 034	2.75	1.52	1.04	0.98	—	—	—	—	—
METSIM	9 607	57%	1.01	1.02	5 504	72%	1.01	1.02	1 507	2.30	0.70	1.01	1.00	1 500	22.90	70.70	0.99	1.01
NESCOG	477	45%	1.04	1.57	216	27%	1.10	1.24	210	2.10	0.80	1.02	1.02	—	—	—	—	—
NAG-FIN	2 052	85%	0.99	1.00	1 649	49%	1.01	1.03	1 716	2.80	0.90	0.99	1.00	1 704	17.70	20.50	1.00	1.01
NTR	7 266	47%	1.01	1.06	3 107	38%	1.01	1.06	2 725	2.40	0.80	1.00	1.01	2 955	18.00	18.10	1.01	1.02
PAGE-CARDIA	—	—	—	—	—	—	—	—	685	15.18[i]	10.65[i]	1.02	NA	—	—	—	—	—
QIMR	—	—	—	—	—	—	—	—	4 409	3.70	1.20	1.05	1.05	4 193	16.80	11.50	1.04	1.04
SardiNIA	5 459	39%	1.00	1.03	2 105	54%	1.01	1.01	2 105	2.50	1.20	1.01	1.03	2 057	21.00	40.50	1.00	1.01
UKB	448 196	45%	1.56	1.07	199 612	17%	1.21	1.06	143 301	2.70	0.87	1.24	1.10	147 551	17.19	11.46	1.20	1.08
NINDS SiGN	3 665	43%	1.00	1.03	1 562	37%	1.00	1.10	—	—	—	—	-	—	—	—	—	—
FINRISK	25 097	51%	1.12	1.06	12 361	55%	1.05	1.03	6 762	2.30	0.80	1.04	1.02	12 912	18.15	23.85	1.03	1.01
WLS	8 496	58%	1.00	1.03	4 915	27%	1.00	1.01	4 821	3.24	1.77	1.00	1.01	4 709	18.01	18.53	1.01	1.00
Spit for Science	2 734	24%	1.03	1.01	—	—	—	—	585	1.43	0.51	0.99	0.98	—	—	—	—	—
AMISH-TOPMed	846	30%	1.01	1.15	209	50%	0.94	1.15	123	1.66	0.82	1.02	1.19	222	18.17	11.12	0.90	1.07
CFS-TOPMed	—	—	—	—	228	41%	0.92	0.98	228	2.67	1.24	1.08	0.95	228	17.56	23.38	1.05	1.09
ECLIPSE-TOPMed	—	—	—	—	1 349	39%	1.01	1.01	1 202	3.15	0.99	1.00	1.04	1 353	17.04	16.32	1.00	1.02

Supplementary Table 2 **Phenotypic descriptive statistics for traits: smoking initiation, smoking cessation, the initiation age of regular smoking, and cigarettes per day** (Continued)

Study name	Smoking initiation[a] (N^e=2 669 029)				Smoking cessation[b] (N^e=1 147 272)				Cigarettes per day[c] (N^e=618 489)					Initiation age of regular smoking[d] (N^e=618 541)				
	n	% Ever smoker	MAF> 0.01	0.01≤ MAF> 0.001	*n*	% Current smoker	MAF> 0.01	0.01≤ MAF> 0.001	*n*	Mean	Var	MAF> 0.01	0.01≤ MAF> 0.001	*N*	Mean	Var	MAF> 0.01	0.01≤ MAF> 0.001
GeneSTAR	913	44%	1.06	0.95	403	40%	0.95	1.05	160	2.56	0.86	1.07	1.11	382	17.39	13.02	0.94	0.97
GOLDN-TOPMed	849	28%	1.03	1.00	234	27%	0.97	0.97	117	2.79	2.34	1.01	1.00	197	16.89	17.58	0.95	0.97
Boston-TOPMed	—	—	—	—	64	17%	1.05	1.11	64	3.61	1.04	1.03	0.96	—	—	—	—	—
IPF-TOPMed	425	73%	0.98	0.96	275	11%	1.03	1.05	126	2.88	1.18	1.01	1.02	125	18.42	30.29	1.02	1.00
CHS-TOPMed	2 295	55%	1.03	0.84	1 264	20%	1.04	1.09	1 219	2.70	1.19	0.91	0.81	—	—	—	—	—
HCHS SOL-TOPMed	—	—	—	—	107	62%	1.00	1.04	91	2.01	1.43	0.98	1.05	—	—	—	—	—
HVH-TOPMed	626	47%	1.01	0.99	296	17%	1.00	1.02	—	—	—	—	-	—	—	—	—	—
JHS-TOPMed	48	29%	1.00	1.10	—	—	—	—	—	—	—	—	-	—	—	—	—	—
VTE-TOPMed	348	64%	1.00	0.96	223	61%	1.00	1.00	—	—	—	—	-	—	—	—	—	—
WGHS-TOPMed	113	50%	1.03	1.00	57	23%	1.04	1.00	—	—	—	—	—	—	—	—	—	—
WHI-TOPMed	9 050	50%	1.08	1.49	4 451	15%	1.04	1.27	3,424	2.66	1.37	1.00	1.05	3,545	19.70	15.56	1.02	1.13

[a]Smoking initiation: Binary phenotype with any participant reporting ever being a regular smoker in their life (current or former) coded "2", while any participant who reported never being a regular smoker in their life coded "1".
[b]Smoking cessation: Binary phenotype with current smokers coded as "2", former smokers coded as "1", and never smokers coded as missing.
[c]Cigarettes per day: Defined as the average number of cigarettes smoked per day, either as a current smoker or former smoker. Individuals who either never smoked, or for whom there is no available data (*e.g.*, someone was a former smoker, but for whom former smoking was never assessed) were set to missing.
[d]Initiation age of regular smoking: Age (in years) at which an individual started smoking cigarettes regularly.
[e]Total sample size.
[f]23andMe provided bins and counts within bins. The counts were on a pack-per-day scale, with the following numbers of individuals within each bin ([0, 0.4), 19 607; (0.4, 0.75], 20 148; (0.75, 1], 20 775; (1, 5], 12 852). For this table, these bins were assigned values of one through four.
[g]The deCODE study used the Fagerström categories, labeling them as 0–3, and an average of 0.9 using these bins would thus be about 15 cigarettes per day.
[h]The deCODE study provided a binned age variable. A median of 9 and a mean of 9.5 correspond to roughly 17.5 years of age.
[i]In PAGE-CARDIA study, the values of cigarettes per day were log-transformed.
Abbreviations: EUR, European; MAF, minor allele frequency; Var, variance.

Supplementary Table 3 **Description of samples used in genome-wide association of lung cancer**

Studies and subgroups	Lung cancer cases (*N*=34 065)		Controls (*N*=470 856)	
	n	%	*n*	%
UKB studies (passed QC)	4 799	14.09	414 406	88.01
OncoArray studies (passed QC)	14 803	43.45	12 262	2.61
Published GWAS[a]	14 463	42.46	44 188	9.38
Total	34 065		470 856	
Age (years)				
≤50	3 377	10.92	110 983	23.89
>50	27 559	89.08	353 530	76.11
Sex				
Male	20 744	60.89	217 395	46.69
Female	13 322	39.11	248 258	53.31
Smoking status				
Never	2 935	9.68	168 098	38.45
Ever	27 395	90.32	269 101	61.55
Histology[b]				
Adenocarcinoma	11 545	33.89	—	—
Squamous cell carcinoma	8 044	23.61	—	—
Small cell carcinoma	2 977	8.74	—	—

[a]Previous GWAS include IARC, MDACC, SLRI, ICR, Harvard, ATBC, CPS-Ⅱ, German, and deCODE studies[1–3].
[b]The subjects for whom histological types were not provided (33.75%) belong to other subtypes, such as large cell carcinoma, non-small cell lung cancer, NOS, mixed histology, and unknown.
Abbreviations: ATBC, Alpha-Tocopherol, Beta-Carotene Cancer Prevention study; CPS-Ⅱ, Cancer Prevention Study Ⅱ nutrition cohort; GWAS, Genome-wide Association Study; IARC, International Agency for Research on Cancer; ICR, Institute of Cancer Research; MDACC, MD Anderson Cancer Center; NOS, not otherwise specified; QC, quality control; SLRI, Samuel Lunenfeld Research Institute; UKB, UK Biobank.

Supplementary Table 4 **SNPs associated with hypothyroidism at the genome-wide level of significance**

SNP	Alt	Ref	*β*	SE	*P*	*F*
rs1001484	G	A	−0.074 3	0.012 4	1.93E−09	36.046 3
rs10085063	T	C	0.075 3	0.012 2	7.16E−10	37.975 4
rs1020812	C	T	0.075 2	0.012 3	1.06E−09	37.212 8
rs10283746	C	T	−0.075 9	0.010 7	1.28E−12	50.358 2
rs1042511	G	T	−0.074 5	0.012 3	1.26E−09	36.881 9
rs10491723	A	G	0.064 2	0.010 6	1.26E−09	36.881 8
rs1057373	A	C	−0.086 7	0.015 2	1.25E−08	32.410 8
rs10748781	A	C	−0.071 9	0.011 1	9.39E−11	41.945 2
rs10758593	A	G	0.064 3	0.010 6	1.41E−09	36.653 8
rs10820727	A	G	−0.094 9	0.016 4	6.85E−09	33.577 9
rs10929817	C	T	−0.066 5	0.010 9	8.65E−10	37.606 8
rs1122326	C	A	0.069 4	0.012 5	2.6E−08	30.989 0
rs11255795	A	G	−0.088 6	0.015 9	2.7E−08	30.913 0
rs113119046	A	C	−0.122 8	0.022 4	4.48E−08	29.929 9
rs113409167	C	T	0.077 2	0.012 9	2.17E−09	35.816 3
rs114243257	T	C	0.118 3	0.017 2	5.27E−12	47.584 5

Supplementary Table 4 **SNPs associated with hypothyroidism at the genome-wide level of significance** (Continued)

SNP	Alt	Ref	*β*	SE	*P*	*F*
rs11507946	T	A	−0.154 9	0.028 1	3.44E−08	30.440 7
rs115606678	A	G	0.170 6	0.020 1	2.31E−17	71.860 2
rs11571297	C	T	−0.110 8	0.011 0	6.5E−24	101.689 7
rs117892843	T	C	−0.156 0	0.025 2	5.64E−10	38.441 6
rs11789701	C	G	0.103 5	0.013 2	4.71E−15	61.378 7
rs11964080	A	G	−0.148 5	0.021 1	2.06E−12	49.426 4
rs1203924	A	G	0.079 4	0.013 1	1.56E−09	36.451 8
rs12175489	A	G	−0.146 4	0.014 9	9.74E−23	96.327 5
rs12202730	T	C	−0.127 9	0.022 6	1.42E−08	32.164 6
rs12236690	C	T	0.070 1	0.012 7	3.72E−08	30.288 3
rs12378800	A	G	−0.157 9	0.024 7	1.6E−10	40.899 8
rs1281944	C	T	0.228 5	0.027 2	4.76E−17	70.433 7
rs1317983	C	T	0.096 0	0.011 5	5.52E−17	70.140 9
rs1351283	G	A	0.104 6	0.010 9	5.75E−22	92.811 4
rs141686764	G	A	−0.235 7	0.039 9	3.38E−09	34.949 8
rs148181670	G	A	−0.263 8	0.047 0	2.03E−08	31.462 6
rs17008423	T	C	−0.126 3	0.016 7	3.38E−14	57.501 7
rs17020067	C	T	−0.057 9	0.010 6	4.01E−08	30.144 9
rs17020127	G	A	0.203 1	0.016 1	2.53E−36	158.400 4
rs1704999	G	A	0.094 3	0.016 4	9.91E−09	32.858 7
rs171329	A	G	−0.069 5	0.011 1	3.56E−10	39.341 6
rs17364832	G	T	0.074 0	0.011 6	1.67E−10	40.823 3
rs1861630	C	T	0.070 2	0.012 2	9.06E−09	33.033 2
rs206018	G	C	−0.070 5	0.012 3	9.12E−09	33.020 5
rs2071401	T	G	−0.099 2	0.017 1	6.57E−09	33.658 2
rs2248372	A	G	−0.090 0	0.010 9	1.35E−16	68.379 5
rs244687	G	A	−0.070 9	0.012 1	4.37E−09	34.453 0
rs2596443	G	A	0.089 6	0.011 0	3.21E−16	66.672 1
rs28559870	T	C	−0.098 7	0.012 0	2.29E−16	67.334 2
rs2859072	A	G	−0.086 3	0.010 7	6.56E−16	65.260 7
rs28746955	C	A	0.113 5	0.013 6	8.16E−17	69.370 9
rs28780083	G	A	−0.117 7	0.018 8	3.9E−10	39.162 8
rs3008034	C	T	−0.083 4	0.012 0	4.16E−12	48.048 7
rs3016013	A	G	0.088 5	0.011 5	1.5E−14	59.096 4
rs3088168	C	T	0.061 2	0.010 9	2.23E−08	31.284 3
rs3116964	T	C	−0.097 0	0.011 1	2.74E−18	76.068 7
rs3134928	T	C	−0.076 8	0.012 0	1.55E−10	40.968 8
rs35149168	C	T	−0.103 9	0.017 4	2.12E−09	35.859 3
rs35956754	C	G	0.093 8	0.010 9	5.26E−18	74.781 5
rs3806155	T	A	0.185 3	0.023 8	7.7E−15	60.411 3
rs429608	A	G	−0.093 6	0.016 4	1.05E−08	32.742 1

Supplementary Table 4 **SNPs associated with hypothyroidism at the genome-wide level of significance** (Continued)

SNP	Alt	Ref	*β*	SE	*P*	*F*
rs4381184	C	A	0.078 0	0.011 8	3.74E−11	43.745 2
rs471081	T	A	0.068 4	0.011 2	9.99E−10	37.327 0
rs4713466	T	C	0.127 9	0.018 6	6.42E−12	47.198 1
rs55855422	A	G	−0.074 1	0.011 8	3.92E−10	39.151 1
rs6550597	A	G	0.065 6	0.011 2	5.43E−09	34.029 4
rs657718	G	A	0.064 3	0.010 6	1.3E−09	36.816 6
rs6724073	C	T	−0.067 9	0.011 0	6.05E−10	38.305 5
rs684231	A	G	−0.087 6	0.012 1	4.36E−13	52.475 4
rs6917212	G	C	0.136 1	0.011 4	5.29E−33	143.209 1
rs7045481	C	T	0.062 5	0.010 9	9.09E−09	33.027 1
rs715299	G	T	0.131 1	0.012 5	6.38E−26	110.850 6
rs72753522	T	C	0.099 7	0.018 0	2.85E−08	30.806 3
rs74302019	T	C	0.066 9	0.012 0	2.23E−08	31.284 0
rs743399	T	C	−0.120 2	0.011 2	8.56E−27	114.832 1
rs74714424	G	T	0.189 4	0.024 4	8.73E−15	60.163 9
rs7525945	C	T	−0.079 0	0.012 9	9.64E−10	37.397 2
rs75319095	C	T	0.088 4	0.014 6	1.44E−09	36.614 2
rs76293252	G	A	−0.149 0	0.025 1	3.05E−09	35.152 2
rs77130284	T	C	0.079 4	0.013 4	3.15E−09	35.087 7
rs7851498	C	T	−0.100 0	0.014 1	1.19E−12	50.495 4
rs7871887	C	T	−0.117 5	0.013 7	9.36E−18	73.642 4
rs7873463	C	A	−0.079 1	0.011 3	2.68E−12	48.909 0
rs7972173	T	A	−0.083 8	0.013 2	2.59E−10	39.962 8
rs897586	A	G	−0.065 8	0.011 4	8.11E−09	33.249 2
rs925488	A	G	0.205 2	0.011 2	6.81E−75	335.278 5
rs9260610	C	T	−0.081 1	0.014 5	2.2E−08	31.310 6
rs9268848	A	G	0.168 7	0.010 6	1.95E−57	255.157 7
rs9273067	A	C	0.144 8	0.013 9	1.74E−25	108.862 7
rs9273401	G	A	0.181 5	0.016 1	2.51E−29	126.404 5
rs9277000	T	C	0.105 8	0.017 5	1.46E−09	36.589 1
rs9373363	G	A	−0.065 1	0.011 2	5.97E−09	33.843 7
rs9497965	T	C	0.068 2	0.011 2	1.15E−09	37.054 7

Abbreviations: Alt, alternative allele; Ref, reference allele; SE, standard error; SNP, Single-nucleotide polymorphism.

Supplementary Table 5 **SNPs associated with hyperthyroidism at the genome-wide level of significance**

SNP	Alt	Ref	*β*	SE	*P*	*F*
rs1015166	T	C	0.117 6	0.018 3	1.19E−10	41.481 5
rs10947439	T	C	−0.144 4	0.019 2	5.31E−14	56.611 5
rs114322847	T	C	0.366 4	0.054 5	1.81E−11	45.168 7
rs11571297	C	T	−0.142 0	0.019 1	9.04E−14	55.565 9
rs13098877	T	C	0.108 3	0.018 3	3.37E−09	34.957 5
rs1383264	T	A	−0.181 4	0.018 8	4.49E−22	93.300 3

Supplementary Table 5 **SNPs associated with hyperthyroidism at the genome-wide level of significance** (Continued)

SNP	Alt	Ref	*β*	SE	*P*	*F*
rs183661661	G	A	0.262 7	0.035 3	9.7E−14	55.428 1
rs2022082	C	T	−0.129 4	0.021 0	6.74E−10	38.092 8
rs2023472	G	A	−0.121 1	0.018 7	1.05E−10	41.720 4
rs206783	A	C	−0.177 0	0.018 2	2.28E−22	94.642 4
rs2072915	A	T	0.115 5	0.021 1	4.36E−08	29.983 3
rs210157	T	C	−0.104 1	0.018 3	1.32E−08	32.296 6
rs2114702	A	T	0.126 4	0.021 8	6.27E−09	33.749 8
rs241429	G	A	−0.222 4	0.018 8	3.14E−32	139.670 9
rs2466074	T	C	−0.122 4	0.018 8	6.69E−11	42.606 8
rs2477233	A	T	0.164 7	0.024 1	8.37E−12	46.677 3
rs2523659	G	T	−0.131 2	0.019 4	1.24E−11	45.899 7
rs2523700	C	G	0.239 1	0.019 4	4.66E−35	152.613 7
rs2844795	T	C	−0.125 0	0.018 2	6.89E−12	47.059 1
rs2853940	C	T	−0.147 4	0.019 5	3.55E−14	57.405 9
rs2853950	T	C	−0.123 0	0.019 2	1.63E−10	40.865 4
rs28746879	G	T	−0.239 5	0.041 8	1.03E−08	32.775 4
rs2928170	G	C	0.129 2	0.022 3	6.59E−09	33.653 2
rs3128947	G	A	−0.159 4	0.028 1	1.37E−08	32.232 2
rs3129267	G	C	−0.168 9	0.018 8	2.39E−19	80.890 1
rs3130182	G	A	−0.185 9	0.021 4	3.91E−18	75.366 6
rs3218815	T	C	−0.118 7	0.021 2	2.16E−08	31.342 0
rs3800307	A	T	0.168 1	0.027 2	6.23E−10	38.247 7
rs3869097	T	C	−0.138 7	0.018 8	1.63E−13	54.403 2
rs439303	A	G	−0.128 3	0.019 2	2.56E−11	44.491 4
rs445832	C	T	−0.110 6	0.018 5	2.51E−09	35.529 9
rs453098	A	G	−0.263 8	0.039 0	1.42E−11	45.645 8
rs4822297	T	C	−0.124 9	0.019 0	4.51E−11	43.380 6
rs542418	G	A	0.123 0	0.018 3	1.86E−11	45.111 6
rs557011	T	C	−0.165 6	0.019 5	1.74E−17	72.418 6
rs57652769	T	C	0.168 3	0.019 5	6.66E−18	74.316 0
rs6902116	G	A	0.204 3	0.018 9	2.94E−27	116.950 9
rs6927229	A	T	0.110 2	0.020 0	3.66E−08	30.319 6
rs72653926	A	G	−0.131 8	0.020 0	4.85E−11	43.238 1
rs73647171	T	G	0.366 4	0.062 8	5.49E−09	34.008 4
rs7743415	T	C	−0.210 2	0.018 2	8.56E−31	133.109 6
rs77546841	G	T	0.103 8	0.018 6	2.45E−08	31.104 4
rs885950	C	A	0.102 9	0.018 7	3.46E−08	30.428 5
rs915894	G	T	0.190 0	0.019 1	2.81E−23	98.791 4
rs9266303	T	G	−0.214 9	0.031 4	7.65E−12	46.852 5
rs9274666	G	A	0.119 2	0.018 3	6.83E−11	42.567 3
rs9275601	T	C	0.160 9	0.019 1	4.01E−17	70.773 7
rs9348772	T	G	0.114 4	0.020 3	1.8E−08	31.700 0
rs9470028	C	T	0.298 3	0.053 2	2.1E−08	31.398 4
rs9500927	A	G	0.346 5	0.026 9	5.67E−38	165.950 4
rs9658172	A	G	0.349 6	0.063 4	3.52E−08	30.394 8

Abbreviations: Alt, alternative allele; Ref, reference allele; SE, standard error; SNP, single-nucleotide polymorphism.

Supplementary Table 6 **SNPs associated with FT4 at the genome-wide level of significance**

SNP	Alt	Ref	*β*	SE	*P*	*F*
rs10119187	T	C	0.049 7	0.008 5	4.11E−09	34.188 1
rs10739496	T	C	0.077 7	0.006 8	4.2E−30	130.564 2
rs10818937	T	C	−0.047 5	0.007 0	1.31E−11	46.045 9
rs10946313	T	C	0.045 5	0.006 8	2.28E−11	44.771 8
rs10984606	T	G	−0.039 8	0.006 5	1.17E−09	37.492 1
rs11103374	T	C	0.076 5	0.011 4	2.39E−11	45.031 2
rs113107469	T	C	0.199 6	0.022 0	1E−19	82.314 4
rs11626434	C	G	0.058 3	0.006 9	4.08E−17	71.390 3
rs118033571	T	C	0.168 2	0.026 6	2.46E−10	39.984 2
rs12033572	C	G	0.115 2	0.017 1	1.45E−11	45.385 0
rs12338024	A	G	0.055 5	0.008 5	7.02E−11	42.633 2
rs145007396	A	G	0.087 8	0.014 4	1.16E−09	37.176 1
rs151293889	A	G	0.119 1	0.018 5	1.23E−10	41.445 8
rs1569783	C	G	0.073 1	0.011 8	5.18E−10	38.377 0
rs17185536	T	C	0.072 6	0.008 1	1.93E−19	80.334 7
rs2235544	A	C	0.138 7	0.006 5	4.2E−101	455.329 9
rs225014	T	C	0.053 5	0.006 7	1.83E−15	63.761 4
rs2294511	A	T	−0.046 5	0.007 4	4.27E−10	39.485 9
rs4149056	T	C	−0.050 6	0.008 9	1.34E−08	32.323 7
rs4842131	T	C	−0.103 7	0.007 5	7.68E−44	191.176 7
rs4954192	T	C	−0.040 9	0.007 1	8.38E−09	33.184 1
rs56069042	A	G	0.106 1	0.018 6	1.16E−08	32.539 1
rs61548990	A	G	−0.039 3	0.006 7	5.4E−09	34.406 1
rs61774852	T	C	0.119 6	0.016 4	3.08E−13	53.183 2
rs67583169	C	G	0.061 3	0.009 5	9.99E−11	41.636 5
rs6785807	A	G	−0.059 0	0.009 3	2.47E−10	40.247 4
rs6855450	T	C	0.116 4	0.011 5	2.76E−24	102.449 6
rs72664111	T	C	−0.100 8	0.014 6	4.31E−12	47.666 7
rs72783371	A	C	0.067 1	0.012 1	2.77E−08	30.752 1
rs73405691	A	G	0.055 6	0.009 8	1.61E−08	32.188 3
rs74510493	T	C	−0.151 0	0.027 0	2.31E−08	31.277 1
rs7530434	T	C	−0.098 5	0.013 2	9.12E−14	55.683 3
rs8063103	C	G	−0.052 2	0.009 2	1.61E−08	32.193 3
rs951366	T	C	0.036 7	0.006 7	4.39E−08	30.004 2
rs10119187	T	C	0.049 7	0.008 5	4.11E−09	34.188 1

Abbreviations: Alt, alternative allele; FT4, free thyroxine; Ref, reference allele; SE, standard error; SNP, single-nucleotide polymorphism.

Supplementary Table 7 SNPs associated with TSH at the genome-wide level of significance

SNP	Alt	Ref	*β*	SE	*P*	*F*
rs10186921	T	C	0.040 4	0.004 7	3.13E−18	74.804 8
rs10415188	A	G	0.038 4	0.005 3	2.21E−13	52.885 7
rs10421676	A	G	−0.027 3	0.004 8	1.05E−08	32.719 9
rs1042678	A	G	0.048 7	0.004 6	1.99E−26	113.583 5
rs1045774	A	G	−0.032 3	0.004 7	3.87E−12	47.815 8
rs10491551	A	G	−0.054 3	0.008 3	7.59E−11	42.526 0
rs10735341	A	G	−0.046 3	0.006 9	1.76E−11	44.960 0
rs10748781	A	C	−0.058 4	0.004 8	6.2E−35	149.731 2
rs10799824	A	G	−0.113 9	0.006 3	4.96E−73	327.300 2
rs10814915	T	C	0.051 7	0.004 6	1.33E−29	128.008 4
rs10878986	T	C	0.026 9	0.004 9	2.4E−08	30.458 2
rs10948097	A	G	−0.057 3	0.007 8	1.95E−13	53.704 4
rs11159482	T	C	0.086 2	0.010 0	5.45E−18	75.031 7
rs111940983	A	G	0.091 4	0.011 9	1.61E−14	59.183 8
rs11207703	T	C	0.033 8	0.005 9	8.04E−09	32.935 2
rs11207705	A	T	−0.038 4	0.006 7	1.16E−08	32.828 9
rs114181608	T	C	0.116 9	0.012 6	1.52E−20	86.192 2
rs114285740	C	G	0.106 8	0.016 7	1.26E−10	41.127 9
rs114860629	T	C	0.110 1	0.014 0	4.06E−15	61.730 3
rs115363355	A	C	0.136 2	0.018 7	3.02E−13	53.137 5
rs115672787	C	G	−0.048 0	0.008 4	1.08E−08	32.434 6
rs11576851	A	G	−0.032 6	0.004 8	5.83E−12	46.657 5
rs1157994	A	G	−0.098 9	0.013 4	1.58E−13	54.441 1
rs11588526	T	C	0.028 9	0.005 0	8.74E−09	33.735 4
rs11592436	C	G	−0.043 2	0.006 0	7.43E−13	51.993 1
rs116909374	T	C	−0.169 2	0.014 9	7.94E−30	128.483 8
rs116956554	A	G	0.047 1	0.006 8	3.49E−12	47.926 4
rs117043818	A	G	−0.094 8	0.016 1	4.71E−09	34.475 0
rs11732089	T	C	0.117 6	0.005 8	6.77E−93	412.807 1
rs117512183	T	C	0.070 7	0.012 6	1.9E−08	31.526 6
rs11755845	T	C	−0.080 4	0.005 3	1.7E−52	231.839 6
rs117573132	T	C	−0.073 7	0.011 4	8.83E−11	41.993 4
rs117764941	A	G	−0.256 7	0.026 7	7.76E−22	92.389 5
rs11794523	C	G	−0.072 6	0.011 6	4.06E−10	39.332 0
rs118039499	A	C	0.181 2	0.016 9	6.3E−27	115.553 3
rs11926459	T	C	0.034 0	0.004 6	1.47E−13	55.362 4
rs12027702	T	G	0.067 7	0.005 2	3.95E−39	170.891 0
rs1203952	A	G	0.060 8	0.005 5	1.26E−28	122.942 5
rs12120601	T	C	0.033 4	0.005 8	8.36E−09	33.298 6
rs12123817	A	G	−0.064 5	0.011 2	7.49E−09	33.343 9
rs12151527	C	G	−0.040 0	0.006 8	5.2E−09	34.566 3

Supplementary Table 7 **SNPs associated with TSH at the genome-wide level of significance** (Continued)

SNP	Alt	Ref	*β*	SE	*P*	*F*
rs12284404	A	G	−0.067 9	0.005 1	2.72E−41	178.847 1
rs12523579	C	G	−0.054 4	0.004 9	1.02E−28	124.565 4
rs12590163	T	C	0.038 7	0.004 7	1.53E−16	68.641 8
rs12743883	A	G	−0.031 1	0.004 8	1E−10	42.462 7
rs12893151	A	C	−0.055 6	0.005 8	6.7E−22	92.274 6
rs12942923	A	G	−0.042 9	0.004 6	5.89E−21	88.139 8
rs12989118	A	G	0.042 2	0.006 2	1.07E−11	46.413 8
rs1317983	T	C	−0.099 4	0.005 1	2.37E−86	383.279 0
rs13189385	T	C	0.058 8	0.009 8	2.65E−09	35.636 4
rs13329353	T	C	0.057 0	0.004 9	1.04E−31	136.757 0
rs1346490	A	C	−0.040 0	0.004 8	5.73E−17	70.243 6
rs1373851	T	C	−0.061 1	0.010 6	6.09E−09	33.473 3
rs141530304	A	G	−0.093 9	0.016 5	1.28E−08	32.582 4
rs141735698	A	T	0.097 4	0.017 6	2.67E−08	30.740 3
rs142848043	T	C	0.133 5	0.018 3	2.94E−13	53.345 7
rs144787326	A	G	0.081 9	0.014 5	1.81E−08	31.811 0
rs145240171	T	C	0.165 5	0.026 5	4.55E−10	38.994 0
rs146625818	T	G	−0.127 2	0.020 6	7.27E−10	38.077 1
rs149583491	C	G	−0.117 4	0.017 8	4.01E−11	43.645 8
rs1536699	T	C	−0.071 5	0.011 3	2.19E−10	40.239 1
rs17020127	A	G	−0.112 4	0.008 1	1.9E−43	191.441 0
rs17121437	T	C	−0.079 0	0.012 6	3.4E−10	39.363 4
rs1801690	C	G	0.064 8	0.009 7	2.76E−11	44.186 5
rs1861628	A	G	−0.097 0	0.005 2	4.11E−79	350.820 8
rs186822670	A	T	0.153 8	0.012 0	1.5E−37	164.752 1
rs2014911	T	C	−0.040 0	0.005 7	1.47E−12	49.479 6
rs2358900	T	C	0.039 2	0.006 2	2.85E−10	40.049 3
rs2358908	A	G	−0.124 8	0.019 4	1.11E−10	41.404 2
rs2396186	T	C	−0.069 0	0.011 2	7.48E−10	38.158 8
rs2667788	A	G	−0.030 5	0.004 8	1.4E−10	40.840 0
rs2739067	A	G	−0.032 3	0.004 8	9.32E−12	45.802 8
rs2745213	A	G	−0.055 4	0.004 7	1.36E−32	140.665 0
rs2808667	T	C	−0.052 3	0.008 9	4.86E−09	34.254 7
rs2928167	A	G	0.138 0	0.007 0	1.86E−85	387.920 1
rs2983511	C	G	−0.115 5	0.005 0	1.4E−120	538.832 8
rs2993047	A	G	−0.032 7	0.004 7	3.71E−12	49.007 4
rs30233	A	G	−0.025 6	0.004 7	3.29E−08	30.036 3
rs310755	A	G	−0.055 3	0.005 3	1.31E−25	109.679 6
rs334725	A	G	0.159 2	0.010 7	2.94E−50	222.940 9
rs34046483	A	G	0.077 2	0.010 3	5.74E−14	56.659 7
rs34983206	A	G	0.030 8	0.005 4	1.24E−08	32.751 6

Supplementary Table 7 **SNPs associated with TSH at the genome-wide level of significance** (Continued)

SNP	Alt	Ref	*β*	SE	*P*	*F*
rs35064085	A	G	0.031 7	0.005 1	5.45E−10	38.981 7
rs35542350	T	G	0.069 1	0.007 0	4.39E−23	97.261 3
rs35587648	A	G	0.032 3	0.004 7	4.53E−12	47.815 8
rs35835377	A	G	0.079 4	0.005 8	5.68E−43	188.180 0
rs35899127	T	C	−0.051 6	0.008 5	1.7E−09	36.595 1
rs3731890	T	C	0.028 3	0.004 9	7.02E−09	33.711 1
rs3783949	T	G	0.034 0	0.004 5	5.36E−14	57.907 5
rs3848572	T	C	0.039 8	0.005 9	2.12E−11	45.665 9
rs398745	A	C	−0.044 1	0.004 7	3.43E−21	89.134 1
rs4074131	A	G	−0.038 7	0.006 4	1.69E−09	36.595 1
rs4268628	A	G	−0.030 5	0.005 3	6.65E−09	33.363 8
rs4273585	A	G	−0.049 9	0.004 6	1.28E−27	119.250 0
rs4352060	T	C	−0.043 4	0.006 7	1.21E−10	41.934 7
rs4402960	T	G	−0.048 4	0.005 0	1.02E−22	94.619 5
rs4445669	T	C	−0.038 3	0.004 6	4.59E−17	70.251 4
rs4571283	A	G	0.045 1	0.007 6	2.72E−09	35.068 1
rs4704404	T	C	−0.074 4	0.007 7	8.08E−22	92.939 6
rs4711755	C	G	0.028 2	0.005 1	3.25E−08	30.849 0
rs4719486	A	G	−0.027 2	0.004 7	8.44E−09	33.908 2
rs4804413	T	C	0.055 1	0.004 7	1.04E−32	139.145 7
rs4888038	T	C	−0.039 9	0.006 4	5.8E−10	38.899 8
rs4933466	A	G	0.035 0	0.004 8	1.37E−13	53.780 2
rs4980917	A	G	0.063 1	0.010 4	1.07E−09	37.114 2
rs55968805	A	G	−0.107 6	0.011 4	3.47E−21	89.509 8
rs56009477	A	G	0.061 2	0.006 4	1.4E−21	91.517 5
rs56035227	A	G	−0.039 1	0.007 2	4.95E−08	29.411 6
rs56217800	A	T	0.044 7	0.007 8	9.33E−09	32.682 5
rs56228667	T	C	0.098 6	0.006 6	1.12E−49	223.156 4
rs56369010	A	G	0.058 1	0.005 4	1.49E−27	116.542 1
rs56788470	A	G	−0.030 3	0.005 2	4.28E−09	34.231 6
rs56899282	A	C	−0.039 8	0.005 5	3.23E−13	52.681 8
rs57732794	A	G	0.089 5	0.007 4	7.53E−34	145.774 9
rs59381142	A	G	−0.058 8	0.005 6	8.83E−26	110.843 8
rs59862746	T	C	−0.030 4	0.005 6	4.83E−08	29.628 1
rs5997969	T	C	0.026 7	0.004 9	4.37E−08	30.007 0
rs60436694	A	C	0.045 8	0.006 8	1.42E−11	45.317 3
rs6085658	T	C	−0.029 1	0.004 8	1.32E−09	37.176 8
rs61765106	T	G	0.040 1	0.006 7	2.01E−09	35.800 0
rs62174435	A	T	−0.084 2	0.012 6	1.94E−11	44.716 0
rs62242087	T	C	0.056 6	0.009 1	6.13E−10	38.355 7
rs62362610	C	G	0.073 8	0.008 4	1.76E−18	76.672 4

Supplementary Table 7 **SNPs associated with TSH at the genome-wide level of significance** (Continued)

SNP	Alt	Ref	*β*	SE	*P*	*F*
rs62364279	A	C	−0.122 9	0.016 8	2.49E−13	53.804 4
rs62401199	T	C	0.050 4	0.007 8	1.48E−10	41.549 1
rs6574647	A	C	−0.035 1	0.005 3	3.56E−11	44.186 5
rs6721104	A	C	−0.086 6	0.012 5	4.45E−12	48.073 7
rs6724073	T	C	0.052 5	0.005 7	2.48E−20	85.236 3
rs6863296	A	C	−0.057 3	0.010 0	7.78E−09	33.154 3
rs6934837	A	G	0.060 8	0.005 2	2.31E−32	137.831 7
rs700750	A	C	0.033 9	0.004 8	7.9E−13	50.452 9
rs7083359	T	C	−0.031 8	0.005 0	1.42E−10	40.845 5
rs7144481	T	C	0.066 2	0.006 7	3.05E−23	97.568 7
rs722540	T	C	0.128 0	0.016 9	2.61E−14	57.661 5
rs72682433	T	C	−0.041 9	0.007 6	4.22E−08	30.268 3
rs72765147	T	C	−0.105 2	0.011 9	7.01E−19	78.404 7
rs72862280	T	C	−0.092 7	0.016 9	4.08E−08	30.243 0
rs72978712	T	C	−0.043 8	0.006 8	9.42E−11	41.445 8
rs73234178	A	G	−0.064 0	0.005 2	1.55E−34	152.722 1
rs73575083	A	G	0.090 5	0.004 9	2.62E−78	344.744 2
rs74657444	T	C	0.064 4	0.008 4	2.6E−14	58.384 5
rs74805471	A	G	−0.085 1	0.009 6	1.16E−18	77.820 5
rs74888443	T	C	0.060 4	0.010 9	2.7E−08	30.902 1
rs75235317	A	G	0.061 9	0.010 3	1.72E−09	36.426 8
rs75797435	A	T	−0.075 6	0.012 5	1.4E−09	36.636 6
rs76629065	A	G	−0.109 8	0.016 6	3.19E−11	44.006 0
rs76772552	A	G	0.084 2	0.014 3	3.99E−09	34.583 4
rs7714529	A	G	−0.090 6	0.005 1	5.97E−71	318.418 8
rs7715119	A	G	0.096 9	0.007 0	1.05E−43	191.263 3
rs7732130	A	G	0.036 2	0.005 1	1.18E−12	50.834 6
rs77366062	T	G	−0.112 0	0.015 7	1.29E−12	50.635 5
rs7758026	C	G	0.048 1	0.007 2	2.62E−11	44.509 8
rs77949234	A	G	0.112 4	0.016 9	2.38E−11	44.463 0
rs77994712	C	G	0.065 7	0.010 9	1.44E−09	36.563 3
rs7873463	A	C	0.050 4	0.005 2	1.14E−22	94.711 6
rs7966590	A	G	0.038 6	0.005 2	5.93E−14	55.554 2
rs79708723	A	G	0.095 0	0.015 1	3.29E−10	39.423 7
rs8006100	T	C	−0.033 9	0.006 1	2.83E−08	30.958 4
rs8018905	T	C	−0.031 7	0.005 2	7.84E−10	37.468 0
rs80266331	T	C	0.119 9	0.020 9	9.66E−09	32.853 8
rs828562	A	G	0.041 6	0.007 1	5.3E−09	34.251 0
rs925488	A	G	0.058 3	0.004 8	4.95E−34	149.218 8
rs9271687	A	G	−0.067 6	0.010 0	1.22E−11	46.144 9
rs9271776	T	C	−0.062 0	0.009 0	7.96E−12	47.063 7

Supplementary Table 7 **SNPs associated with TSH at the genome-wide level of significance** (Continued)

SNP	Alt	Ref	*β*	SE	*P*	*F*
rs927316	T	C	−0.030 4	0.005 0	7.32E−10	37.328 2
rs9298749	A	C	−0.031 3	0.004 8	5.79E−11	43.010 6
rs9365898	T	C	0.083 5	0.008 3	1.86E−23	100.560 5
rs9497965	T	C	0.040 2	0.004 7	9.73E−18	74.066 0
rs9511147	C	G	0.040 6	0.004 9	5.66E−17	69.382 8
rs9687206	A	G	−0.147 5	0.004 7	1E−200	997.127 4
rs9853146	A	G	−0.030 4	0.004 7	4.83E−11	42.355 9
rs9865818	A	G	0.026 0	0.004 7	3.42E−08	30.982 3
rs9967835	A	G	−0.066 5	0.008 2	7.02E−16	65.366 5

Abbreviations: Alt, alternative allele; Ref, reference allele; SE, standard error; SNP, single-nucleotide polymorphism; TSH, thyroid-stimulating hormone.

Supplementary Table 8 **Genetic risk score (GRS) for the association of thyroid dysfunction and lung cancer risk in the UK Biobank cohort**

Exposure	Population	Cox proportional hazards regression		Logistic regression	
		HR per SD (95% CI)	*P*	OR per SD (95% CI)	*P*
Hypothyroidism-based GRS	Overall[a]	1.07 (1.04, 1.09)	4.61×10^{-6}	1.07 (1.04, 1.09)	5.94×10^{-6}
	Ever-smokers[b]	1.06 (1.04, 1.10)	6.54×10^{-6}	1.07 (1.04, 1.10)	8.55×10^{-6}
	Never-smokers[b]	1.04 (0.96, 1.13)	0.306	1.04 (0.96, 1.13)	0.306
Hyperthyroidism-based GRS	Overall[a]	1.04 (1.01, 1.07)	0.006	1.04 (1.01, 1.07)	0.007
	Ever-smokers[b]	1.04 (1.01, 1.07)	0.015	1.04 (1.01, 1.07)	0.017
	Never-smokers[b]	1.05 (0.97, 1.14)	0.192	1.06 (0.97, 1.14)	0.194

[a]Model adjusted for smoke status, age, sex, BMI, and first 10 principal components.
[b]Model adjusted for age, sex, BMI, and first 10 principal components.
Abbreviations: BMI, body mass index; CI, confidence interval; HR, hazard ratio; OR, odds ratio; SD, standard deviation.

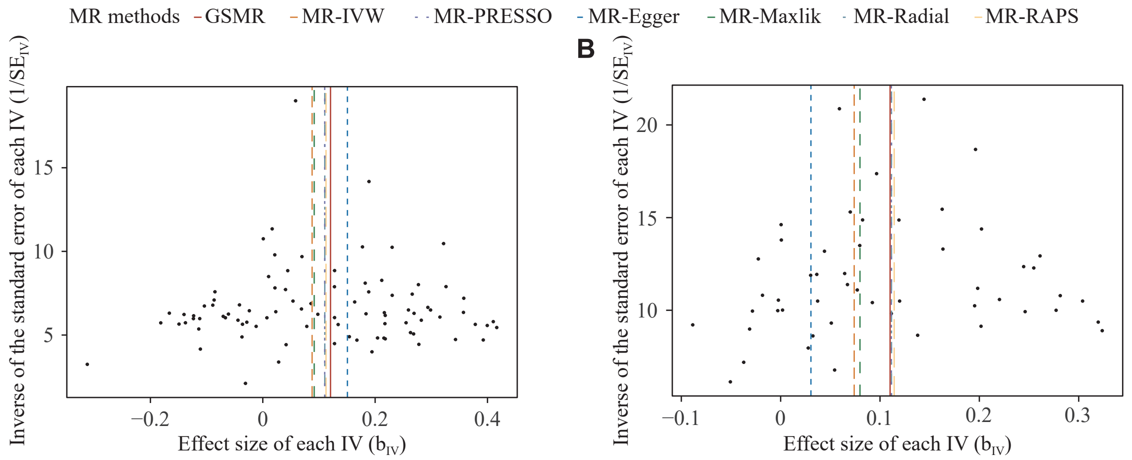

Supplementary Fig. 1 **Funnel plots depicting the causal effect of thyroid dysfunction on lung cancer in the overall population.** The X axis presents the estimate of the causal effect, and the Y axis presents the relevant inverse standard error. Each point represents an individual SNP used as an IV. A: Funnel plot of the causal effect of hypothyroidism on lung cancer in the overall population. B: Funnel plot of the causal effect of hyperthyroidism on lung cancer in the overall population. Abbreviations: GSMR, generalized summary-data-based Mendelian randomization; IV, instrumental variable; MR, Mendelian randomization; MR-Egger, Mendelian randomization with Egger regression; MR-IVW, inverse-variance weighted two-sample Mendelian randomization; MR-Maxlik, Mendelian randomization using maximum-likelihood method; MR-PRESSO, Mendelian randomization pleiotropy residual sum and outlier; MR-Radial, Mendelian randomization with radial regression; MR-RAPS, Mendelian randomization with robust adjusted profile score; SNP, single nucleotide polymorphism.

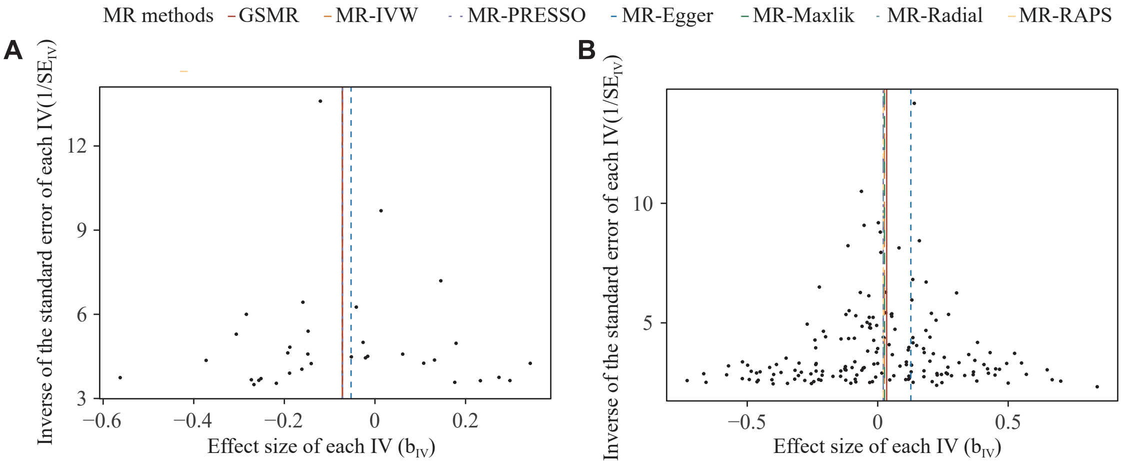

Supplementary Fig. 2 **Funnel plots depicting the causal effect of thyroid function phenotypes on lung cancer in the overall population.** The X axis presents the estimate of the causal effect, and the Y axis presents the relevant inverse standard error. Each point represents an individual SNP used as an IV. A: Funnel plot of the causal effect of FT4 and lung cancer in the overall population. B: Funnel plot of the causal effect of TSH and lung cancer in the overall population. Abbreviations: FT4, free thyroxine; GSMR, generalized summary-data-based Mendelian randomization; IV, instrumental variable; MR, Mendelian randomization; MR-Egger, Mendelian randomization with Egger regression; MR-IVW, inverse-variance weighted two-sample Mendelian randomization; MR-Maxlik, Mendelian randomization using maximum-likelihood method; MR-PRESSO, Mendelian randomization pleiotropy residual sum and outlier; MR-Radial, Mendelian randomization with radial regression; MR-RAPS, Mendelian randomization with robust adjusted profile score; SNP, single nucleotide polymorphism; TSH, thyroid-stimulating hormone.

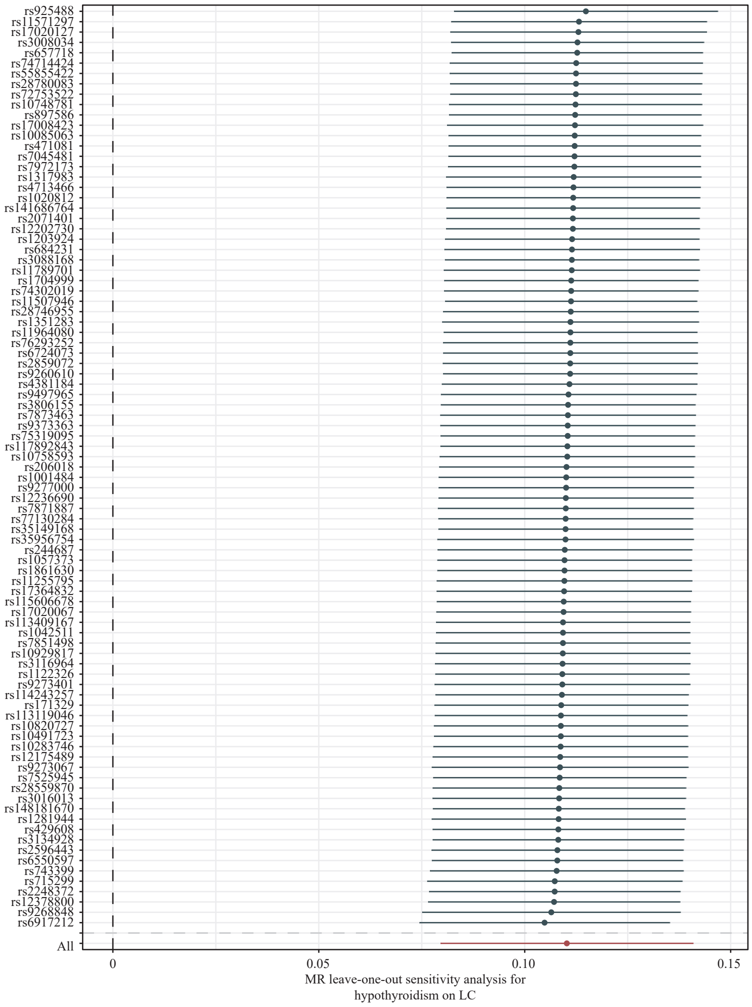

Supplementary Fig. 3 **Causal effects of hypothyroidism on lung cancer using the inverse-variance weighted (IVW) method in the overall population by leave-one-out analysis.** Abbreviations: LC, lung cancer; MR, Mendelian randomization.

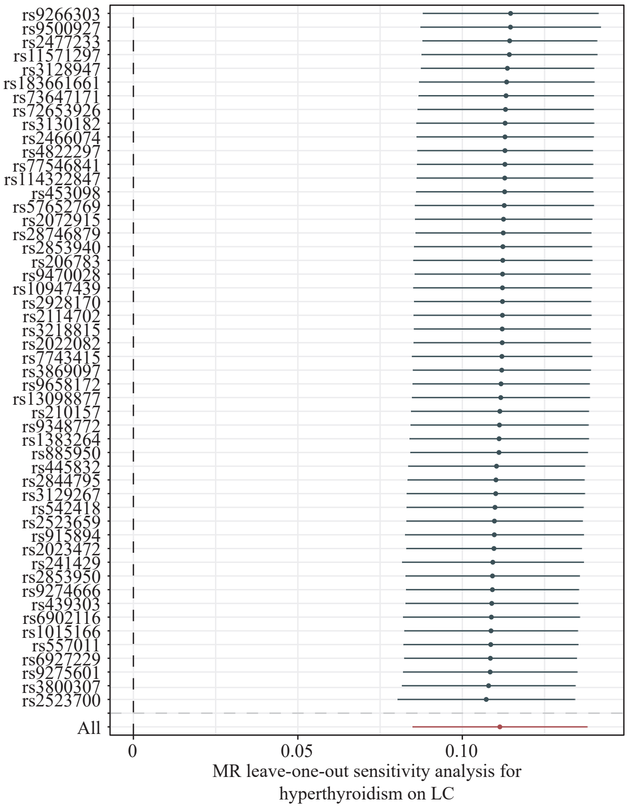

Supplementary Fig. 4 **Causal effects of hyperthyroidism on lung cancer using the inverse-variance weighted (IVW) method in the overall population by leave-one-out analysis.** Abbreviations: LC, lung cancer; MR, Mendelian randomization.

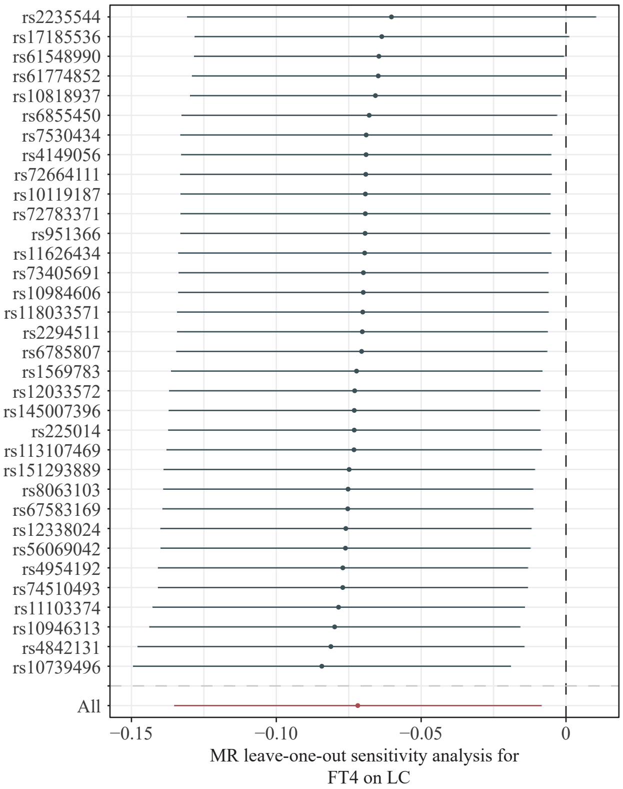

Supplementary Fig. 5 **Causal effects of FT4 on lung cancer using the inverse-variance weighted (IVW) method in the overall population by leave-one-out analysis.** Abbreviations: FT4, free thyroxine; LC, lung cancer; MR, Mendelian randomization.

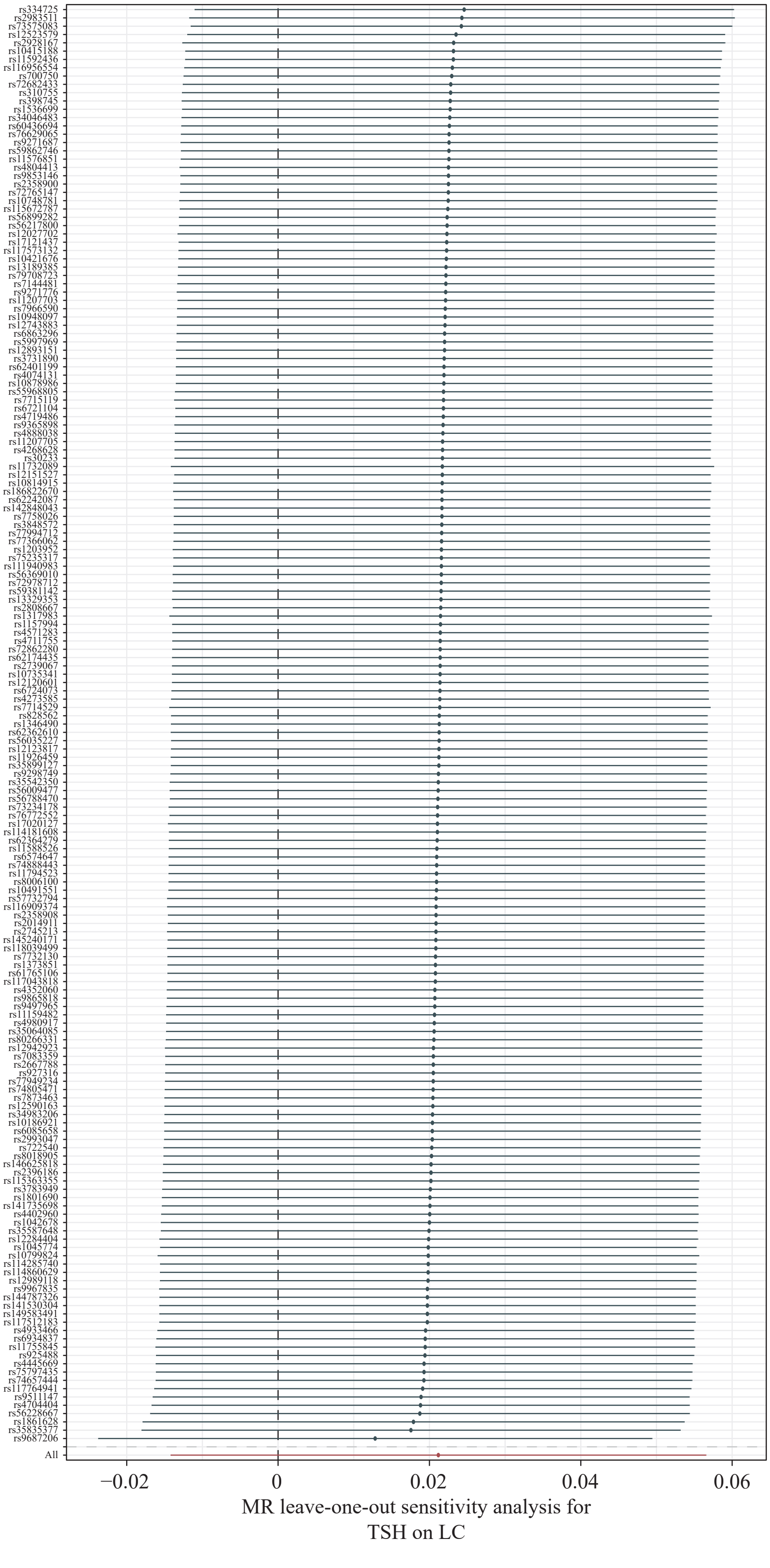

Supplementary Fig. 6 **Causal effects of TSH on lung cancer using the inverse-variance weighted (IVW) method in the overall population by leave-one-out analysis.** Abbreviations: LC, lung cancer; MR, Mendelian randomization; TSH, thyroid-stimulating hormone.

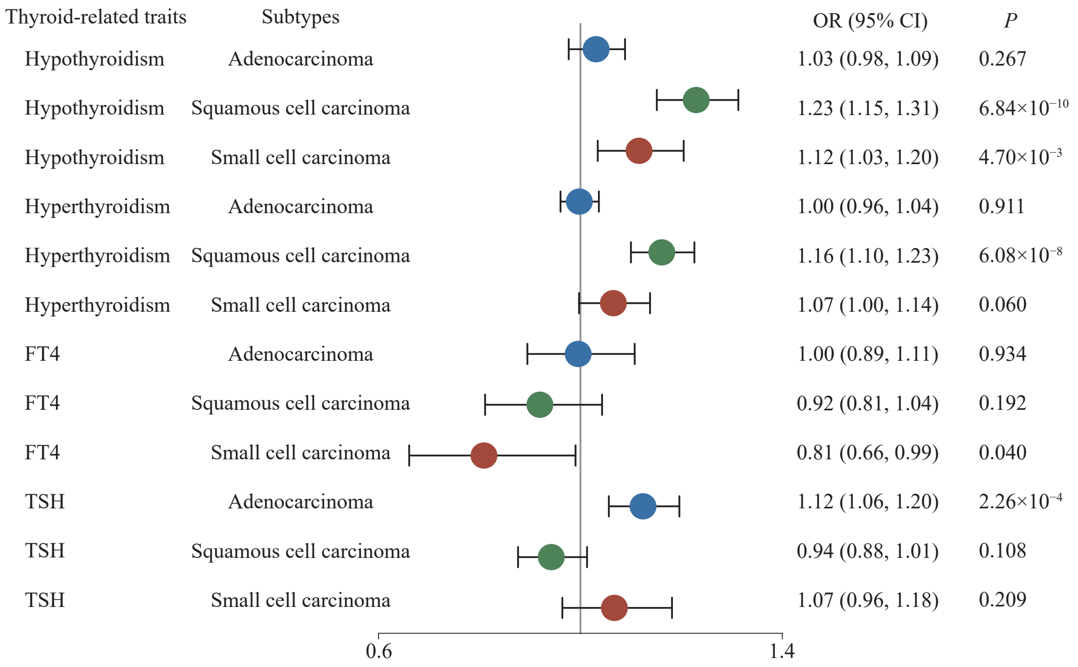

Supplementary Fig. 7 **Forest plots for the estimated causal effects of thyroid-related phenotypes and lung cancer histological subtypes using the random-effects inverse-variance weighted method.**

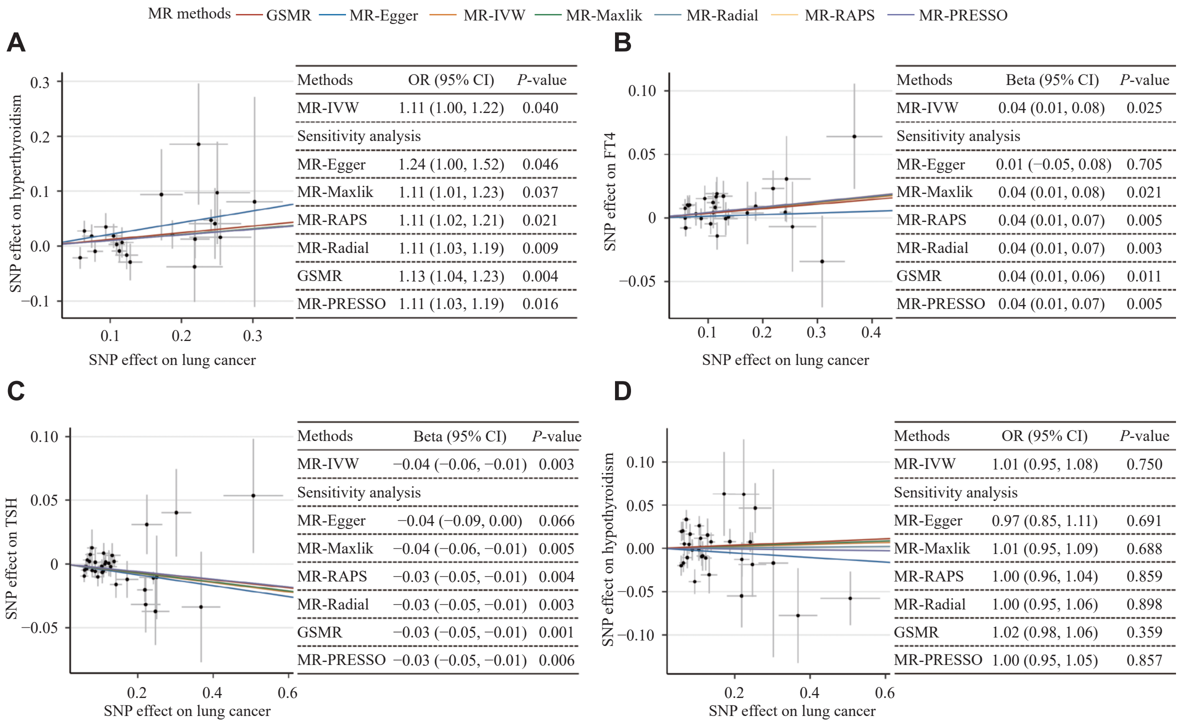

Supplementary Fig. 8 **Scatter plots showing the causal associations between lung cancer risk and thyroid-related phenotypes in the overall population.** Each cross represents an instrumental variable. Error bars indicate 95% CIs. The lines with different colors were estimated by different MR methods; the slope of each line represents the estimated association between lung cancer risk and thyroid-related phenotypes. A: Causal effect between lung cancer and hyperthyroidism using different MR methods. B: Causal effect between lung cancer and FT4 using different MR methods. C: Causal effect between lung cancer and TSH using different MR methods. D: Causal effect between lung cancer and hypothyroidism using different MR methods. Abbreviations: CI, confidence interval; FT4, free thyroxine; GSMR, generalized summary-data-based Mendelian randomization; MR, Mendelian randomization; MR-Egger, Mendelian randomization with Egger regression; MR-IVW, inverse-variance weighted two-sample Mendelian randomization; MR-Maxlik, Mendelian randomization using maximum-likelihood method; MR-PRESSO, Mendelian randomization pleiotropy residual sum and outlier; MR-Radial, Mendelian randomization with radial regression; MR-RAPS, Mendelian randomization with robust adjusted profile score; OR, odds ratio; SNP, single-nucleotide polymorphism; TSH, thyroid-stimulating hormone.

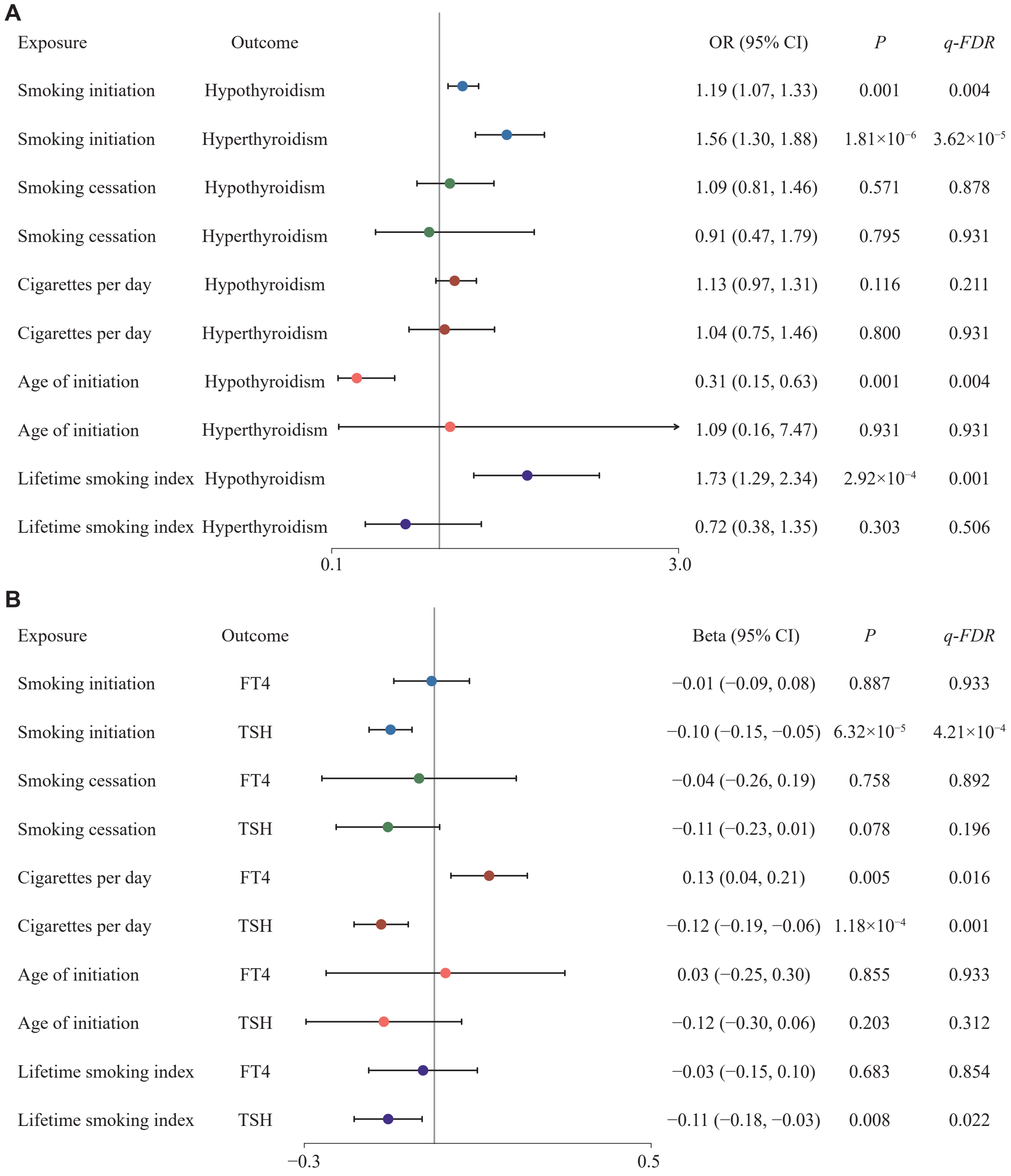

Supplementary Fig. 9 **Forest plots for the estimated causal effects of smoking phenotypes on thyroid-related phenotypes using the random-effects inverse-variance weighted method.** A: Forest plots for the estimated causal effects of smoking phenotypes on thyroid dysfunction. B: Forest plots for the estimated causal effects of smoking phenotypes on thyroid function phenotypes.

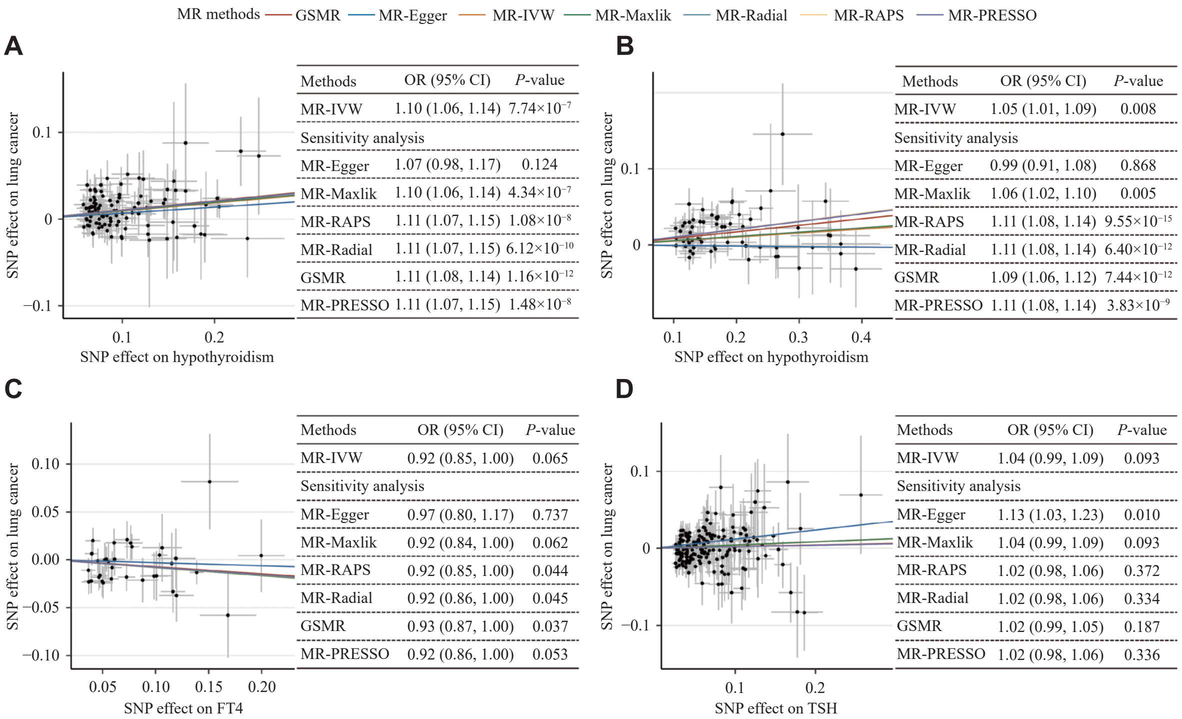

A

Methods	OR (95% CI)	*P*-value
MR-IVW	1.10 (1.06, 1.14)	7.74×10^{-7}
Sensitivity analysis		
MR-Egger	1.07 (0.98, 1.17)	0.124
MR-Maxlik	1.10 (1.06, 1.14)	4.34×10^{-7}
MR-RAPS	1.11 (1.07, 1.15)	1.08×10^{-8}
MR-Radial	1.11 (1.07, 1.15)	6.12×10^{-10}
GSMR	1.11 (1.08, 1.14)	1.16×10^{-12}
MR-PRESSO	1.11 (1.07, 1.15)	1.48×10^{-8}

B

Methods	OR (95% CI)	*P*-value
MR-IVW	1.05 (1.01, 1.09)	0.008
Sensitivity analysis		
MR-Egger	0.99 (0.91, 1.08)	0.868
MR-Maxlik	1.06 (1.02, 1.10)	0.005
MR-RAPS	1.11 (1.08, 1.14)	9.55×10^{-15}
MR-Radial	1.11 (1.08, 1.14)	6.40×10^{-12}
GSMR	1.09 (1.06, 1.12)	7.44×10^{-12}
MR-PRESSO	1.11 (1.08, 1.14)	3.83×10^{-9}

C

Methods	OR (95% CI)	*P*-value
MR-IVW	0.92 (0.85, 1.00)	0.065
Sensitivity analysis		
MR-Egger	0.97 (0.80, 1.17)	0.737
MR-Maxlik	0.92 (0.84, 1.00)	0.062
MR-RAPS	0.92 (0.85, 1.00)	0.044
MR-Radial	0.92 (0.86, 1.00)	0.045
GSMR	0.93 (0.87, 1.00)	0.037
MR-PRESSO	0.92 (0.86, 1.00)	0.053

D

Methods	OR (95% CI)	*P*-value
MR-IVW	1.04 (0.99, 1.09)	0.093
Sensitivity analysis		
MR-Egger	1.13 (1.03, 1.23)	0.010
MR-Maxlik	1.04 (0.99, 1.09)	0.093
MR-RAPS	1.02 (0.98, 1.06)	0.372
MR-Radial	1.02 (0.98, 1.06)	0.334
GSMR	1.02 (0.99, 1.05)	0.187
MR-PRESSO	1.02 (0.98, 1.06)	0.336

Supplementary Fig. 10 **Scatter plots showing the associations between thyroid function phenotypes and lung cancer in ever-smokers.** Each cross represents an instrumental variable. Error bars indicate 95% CIs. The lines with different colors were estimated by different MR methods. The slope of each line represents the estimated association of thyroid function-related phenotypes and lung cancer risk. A: Causal association between hypothyroidism and lung cancer using different MR methods. B: Causal association between hyperthyroidism and lung cancer using different MR methods. C: Causal association between FT4 and lung cancer using different MR methods. D: Causal association between TSH and lung cancer using different MR methods. Abbreviations: CI, confidence interval; FT4, free thyroxine; GSMR, generalized summary-data-based Mendelian randomization; MR, Mendelian randomization; MR-Egger, Mendelian randomization with Egger regression; MR-IVW, inverse-variance weighted two-sample Mendelian randomization; MR-Maxlik, Mendelian randomization using maximum-likelihood method; MR-PRESSO, Mendelian randomization pleiotropy residual sum and outlier; MR-Radial, Mendelian randomization with radial regression; MR-RAPS, Mendelian randomization with robust adjusted profile score; OR, odds ratio; SNP, single-nucleotide polymorphism; TSH, thyroid-stimulating hormone.

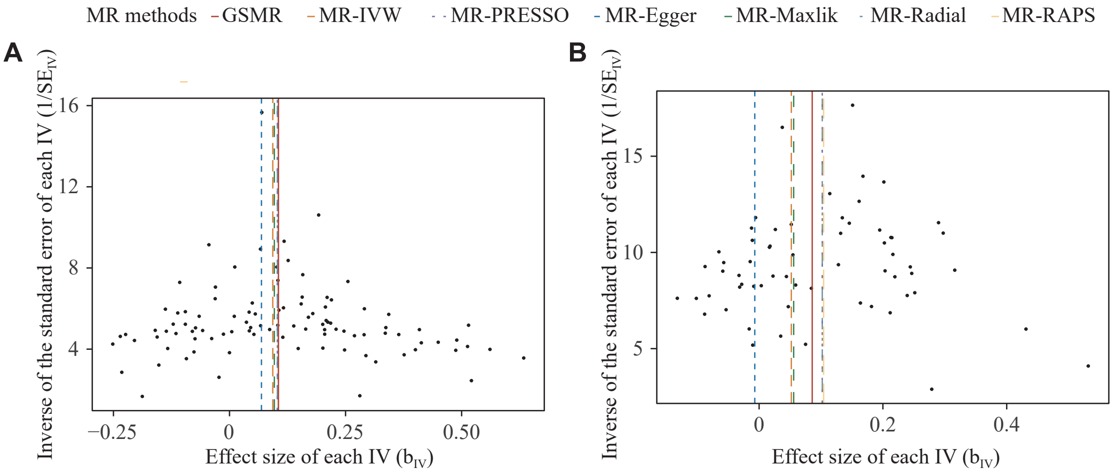

Supplementary Fig. 11 **Funnel plots depicting the causal effect of thyroid dysfunction on lung cancer in ever-smokers.** The X axis presents the estimate of the causal effect, and the Y axis presents the relevant inverse standard error. Each point represents an individual SNP used as an IV. A: Funnel plot of the causal effect of hypothyroidism on lung cancer in ever-smokers. B: Funnel plot of the causal effect of hyperthyroidism on lung cancer in ever-smokers. Abbreviations: GSMR, generalized summary-data-based Mendelian randomization; IV, instrumental variable; MR, Mendelian randomization; MR-Egger, Mendelian Randomization with Egger regression; MR-IVW, Inverse-variance weighted two-sample Mendelian randomization; MR-Maxlik, Mendelian randomization using maximum-likelihood method; MR-PRESSO, Mendelian randomization pleiotropy residual sum and outlier; MR-Radial, Mendelian randomization with radial regression; MR-RAPS, Mendelian randomization with robust adjusted profile score; SNP, single nucleotide polymorphism.

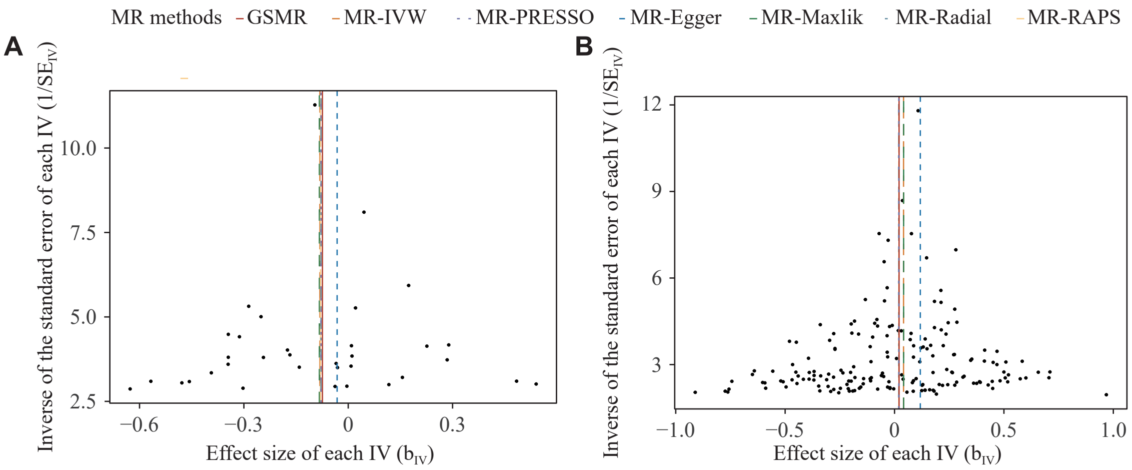

Supplementary Fig. 12 **Funnel plots depicting the causal effect of thyroid function phenotypes on lung cancer in ever-smokers.** The X axis presents the estimate of the causal effect, and the Y axis presents the relevant inverse standard error. Each point represents an individual SNP used as an IV. A: Funnel plot of the causal effect of FT4 and lung cancer in ever-smokers. B: Funnel plot of the causal effect of TSH on lung cancer in ever-smokers. Abbreviations: FT4, free thyroxine; GSMR, generalized summary-data-based Mendelian randomization; IV, instrumental variable; MR, Mendelian randomization; MR-Egger, Mendelian randomization with Egger regression; MR-IVW, inverse-variance weighted two-sample Mendelian randomization; MR-Maxlik, Mendelian randomization using maximum-likelihood method; MR-Radial, Mendelian randomization with radial regression; MR-PRESSO, Mendelian randomization pleiotropy residual sum and outlier; MR-RAPS, Mendelian randomization with robust adjusted profile score; SNP, single nucleotide polymorphism; TSH, thyroid-stimulating hormone.

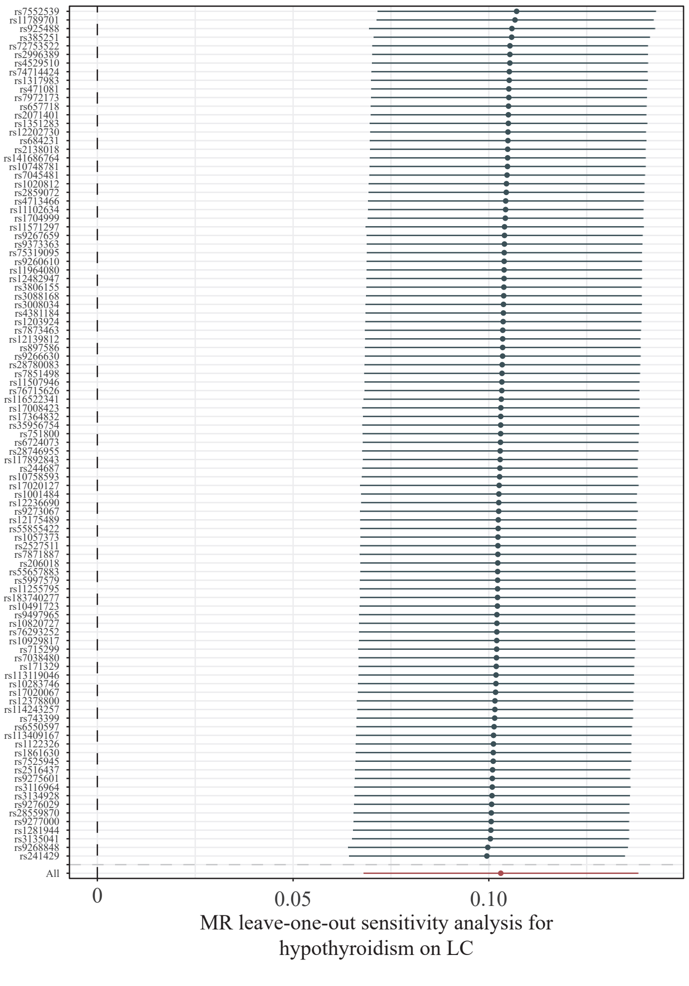

Supplementary Fig. 13 **Causal effects of hypothyroidism on lung cancer using the inverse-variance weighted method in the ever-smoker population by leave-one-out analysis.**

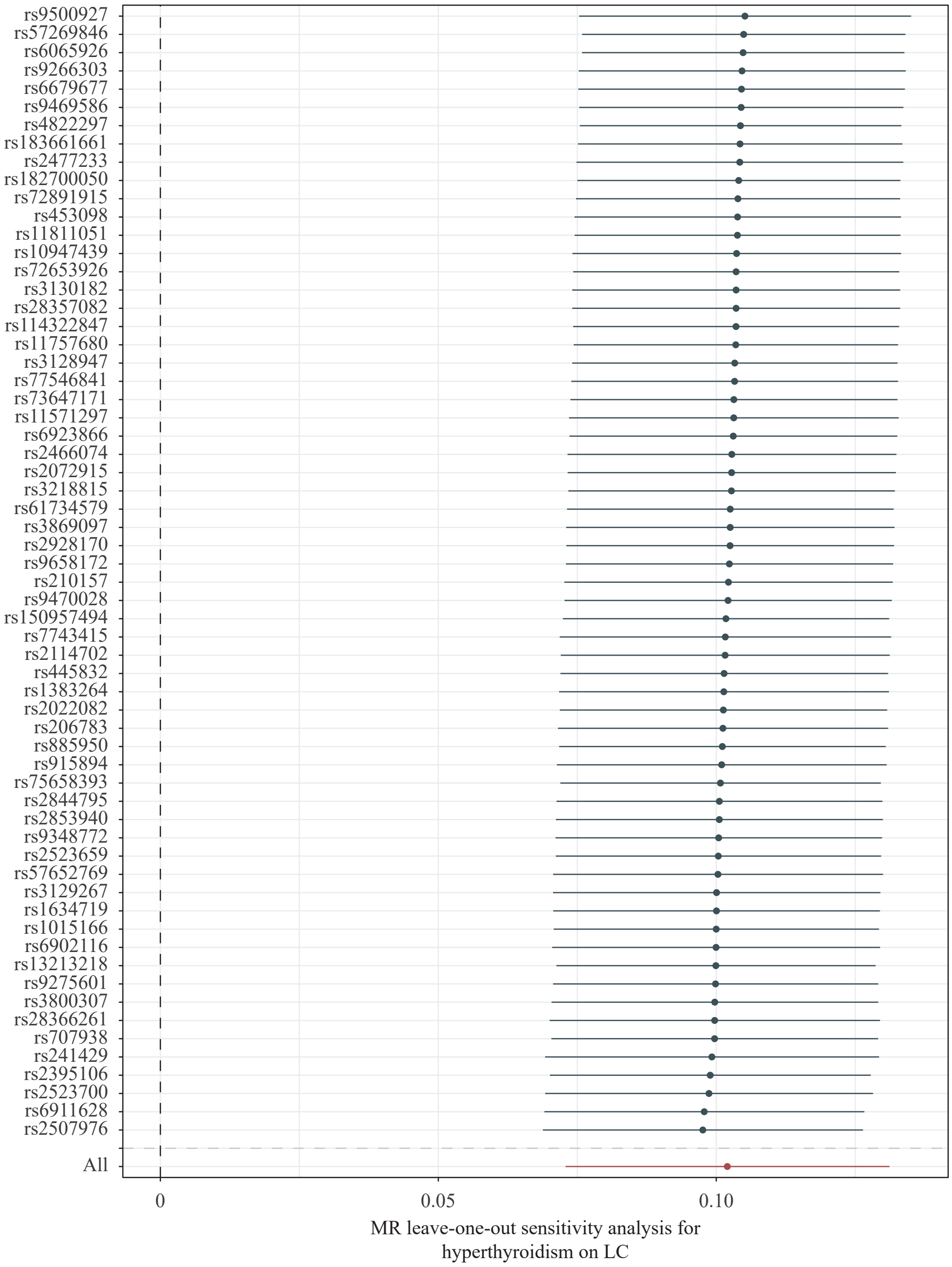

Supplementary Fig. 14 **Causal effects of hyperthyroidism on lung cancer using the inverse-variance weighted method in the ever-smoker population by leave-one-out analysis.**

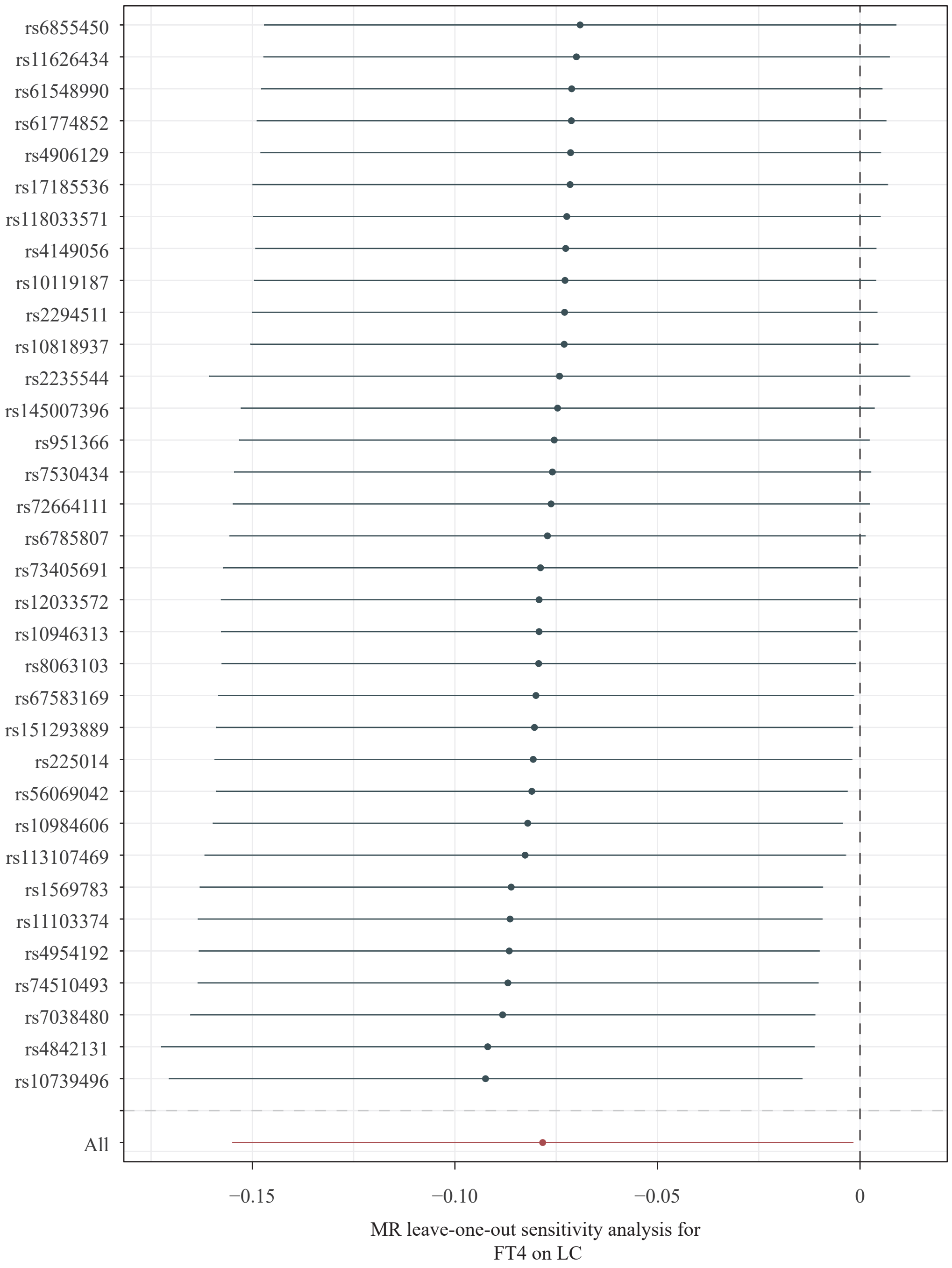

Supplementary Fig. 15 **Causal effects of FT4 on lung cancer using the inverse-variance weighted method in the ever-smoker population by leave-one-out analysis.**

References

[1] Timofeeva MN, Hung RJ, Rafnar T, et al. Influence of common genetic variation on lung cancer risk: Meta-analysis of 14 900 cases and 29 485 controls[J]. *Hum Mol Genet*, 2012, 21(22): 4980–4995.

[2] Wang Y, McKay JD, Rafnar T, et al. Rare variants of large effect in BRCA2 and CHEK2 affect risk of lung cancer[J]. *Nat Genet*, 2014, 46(7): 736–741.

[3] Wang Y, Wei Y, Gaborieau V, et al. Deciphering associations for lung cancer risk through imputation and analysis of 12 316 cases and 16 831 controls[J]. *Eur J Hum Genet*, 2015, 23(12): 1723–1728.
